# A trimethoprim derivative impedes antibiotic resistance evolution

**DOI:** 10.1038/s41467-021-23191-z

**Published:** 2021-05-19

**Authors:** Madhu Sudan Manna, Yusuf Talha Tamer, Ilona Gaszek, Nicole Poulides, Ayesha Ahmed, Xiaoyu Wang, Furkan C. R. Toprak, DaNae R. Woodard, Andrew Y. Koh, Noelle S. Williams, Dominika Borek, Ali Rana Atilgan, John D. Hulleman, Canan Atilgan, Uttam Tambar, Erdal Toprak

**Affiliations:** 1 Green Center for Systems Biology, University of Texas Southwestern Medical Center, Dallas, TX USA; 2 Department of Biochemistry, University of Texas Southwestern Medical Center, Dallas, TX USA; 3 Texas A&M University, College Station, TX USA; 4 Department of Ophthalmology, University of Texas Southwestern Medical Center, Dallas, TX USA; 5 Department of Pediatrics, University of Texas Southwestern Medical Center, Dallas, TX USA; 6 Department of Microbiology, University of Texas Southwestern Medical Center, Dallas, TX USA; 7 Department of Molecular Biophysics, University of Texas Southwestern Medical Center, Dallas, TX USA; 8 Faculty of Engineering and Natural Sciences, Sabanci University, Istanbul, Turkey; 9 Department of Pharmacology, University of Texas Southwestern Medical Center, Dallas, TX USA

**Keywords:** Molecular evolution, Antimicrobials

## Abstract

The antibiotic trimethoprim (TMP) is used to treat a variety of *Escherichia coli* infections, but its efficacy is limited by the rapid emergence of TMP-resistant bacteria. Previous laboratory evolution experiments have identified resistance-conferring mutations in the gene encoding the TMP target, bacterial dihydrofolate reductase (DHFR), in particular mutation L28R. Here, we show that 4’-desmethyltrimethoprim (4’-DTMP) inhibits both DHFR and its L28R variant, and selects against the emergence of TMP-resistant bacteria that carry the L28R mutation in laboratory experiments. Furthermore, antibiotic-sensitive *E. coli* populations acquire antibiotic resistance at a substantially slower rate when grown in the presence of 4’-DTMP than in the presence of TMP. We find that 4’-DTMP impedes evolution of resistance by selecting against resistant genotypes with the L28R mutation and diverting genetic trajectories to other resistance-conferring DHFR mutations with catalytic deficiencies. Our results demonstrate how a detailed characterization of resistance-conferring mutations in a target enzyme can help identify potential drugs against antibiotic-resistant bacteria, which may ultimately increase long-term efficacy of antimicrobial therapies by modulating evolutionary trajectories that lead to resistance.

## Abstract (web summary)

The efficacy of the antibiotic trimethoprim, which inhibits bacterial dihydrofolate reductase (DHFR), is limited by the rapid emergence of resistant bacteria. Here, Manna et al. show that 4’-desmethyltrimethoprim inhibits DHFR and a common TMP-resistant variant, and impedes evolution of antibiotic resistance by selecting against the emergence of this variant.

## Introduction

Antibiotic resistance is a burgeoning public health crisis, with a marked rise in mortality and morbidity associated with antibiotic resistant infections^1,2^. With only a handful of antibiotic target proteins, bacteria are only a few genetic mutations away from becoming completely resistant to several antibiotic molecules. As such, the antibiotic resistance crisis will likely not be solved by developing the next novel antibiotic molecule^3,4^. Therefore, understanding the mechanisms of antibiotic resistance and developing strategies to counteract the evolution of antibiotic resistance will be crucial to combat this public health predicament.

Evolution of antibiotic resistance has been studied at the molecular level for decades with the ultimate goal of devising targeted therapies to impede the evolution of resistance. Targeting evolutionarily common resistance-conferring mutations was previously proposed as a promising strategy to impede evolution of resistance based on computer simulations^5–7^. However, to the best of our knowledge, there has been no biological validation of this strategy. We hypothesized that by comprehensively understanding the molecular evolution of antibiotic resistance in pathogenic bacteria, we would be able to identify and target common mutations and subsequently impede the evolution of antibiotic resistance. To test this hypothesis, we utilized *Escherichia coli* (*E. coli*), a common gram-negative pathogenic bacteria, and trimethoprim (TMP), an antibiotic widely used to treat a variety of *E. coli* infections such as urinary tract infections^8,9^, but limited in its efficacy given the rapidity with which TMP resistance develops^10^.

**Fig. 1:**
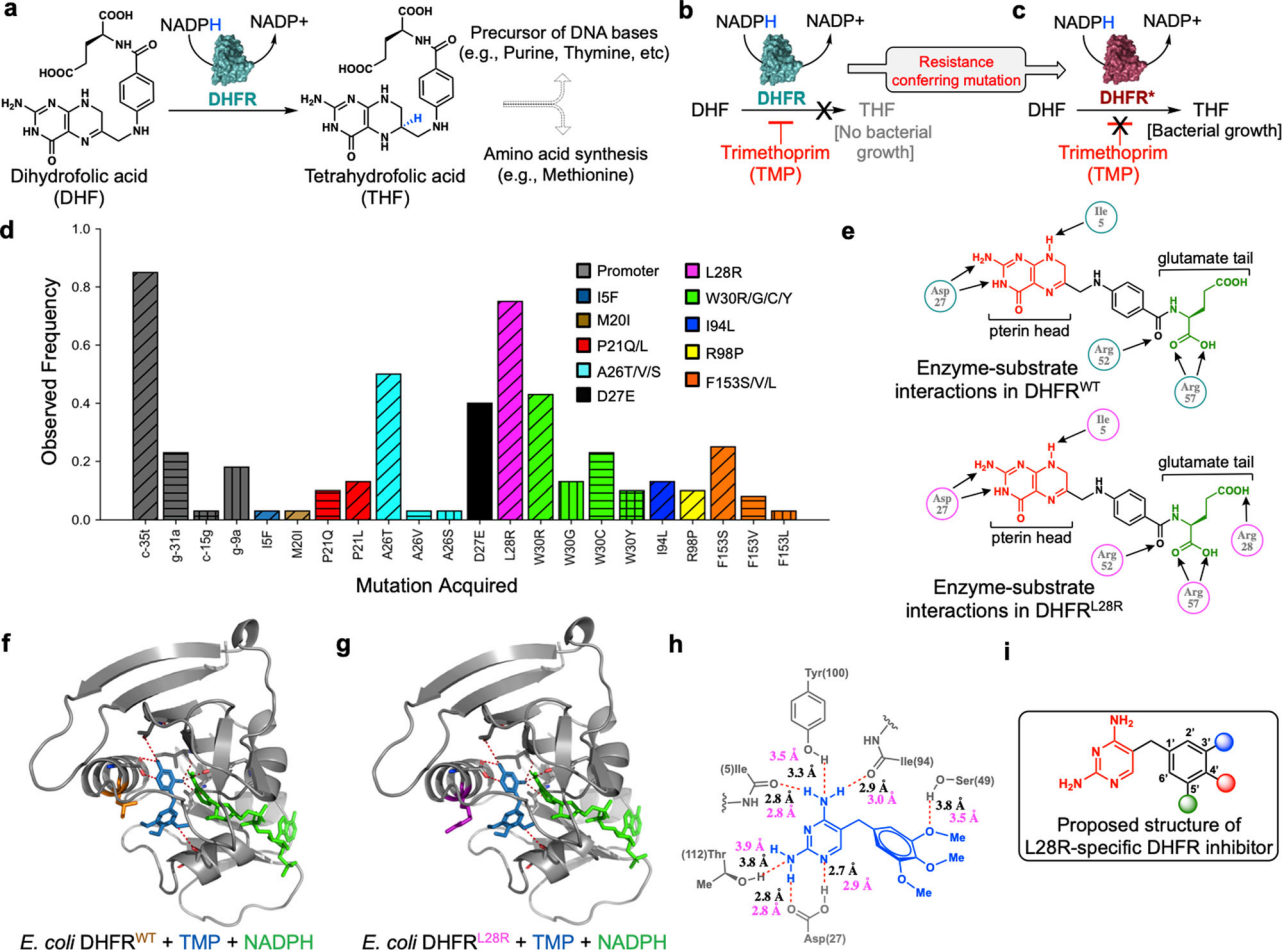
L28R is a coding mutation in *E. coli* DHFR with a unique resistance-conferring mechanism. **a** DHFR is an essential enzyme with a central role in the biosynthesis of nucleotides and amino acids. **b** Trimethoprim (TMP) is a bacteriostatic antibiotic molecule that competitively inhibits DHFR activity. **c** Resistance-conferring DHFR mutations that reduce TMP affinity are responsible for the evolution of TMP resistance in *E. coli*. As TMP is a competitive inhibitor of DHFR, resistance-conferring mutations typically reduce both TMP and substrate (DHF) binding affinities. **d** Frequencies of resistance-conferring DHFR mutations from 40 independent *E. coli* populations evolved in the morbidostat under TMP selection [7 populations from this study (Fig. 4) and 33 populations from our previous studies^7,16^]. L28R is one of the most commonly observed DHFR mutation in laboratory evolution experiments. Different colors on the bar plot are used to represent mutated residues. Stripes with different directionalities are used to distinguish frequency of different amino acid replacements in the same residue. **e** Unlike other mutations, L28R indirectly increases TMP resistance (higher *K*_i_) by increasing substrate affinity (lower *K*_m_) due to additional interactions with the glutamate tail (green) of DHF (bottom) in comparison with wild-type (top). **f** X-Ray crystal structure of DHFR^WT^ (6XG5, resolution: 1.9 Å) bound to TMP drug (blue) and NADPH co-factor (green). **g** X-Ray crystal structure of DHFR^L28R^ (6XG4, resolution: 2.1 Å) bound to TMP drug (blue) and NADPH co-factor (green). Omit difference electron density maps for trimethoprim in both structures are provided in Supplementary Fig. 10. **h** Interaction distances (Å) between DHFR residues (gray) and TMP (blue) for DHFR^WT^ (black) DHFR^L28R^ (magenta). **i** Proposed structure of L28R-specific DHFR inhibitor with modifications (colored spheres) in the hydrophobic tail of TMP while preserving its polar head (red).

TMP mediates its anti-bacterial effect by targeting bacterial dihydrofolate reductase (DHFR), a ubiquitous enzyme found in all organisms and thus also an important target for cancer and autoimmune disorders (Fig. 1a, b)^11–15^. DHFR catalyzes the reduction of 5,6-dihydrofolic acid (DHF) to 5,6,7,8-tetrahydrofolic acid (THF) by enantiospecific hydride transfer from NADPH cofactor (Fig. 1a). THF and its derivatives are essential precursors for the biosynthesis of DNA bases and amino acids^11^. We previously carried out several laboratory evolution experiments with *E. coli* and TMP using the morbidostat, an automated continuous culture device we developed to study antibiotic resistance under nearly constant selection pressure induced by antibiotics^16,17^. Strikingly, we found that evolution of TMP resistance in *E. coli* consistently proceeds through stepwise acquisition of multiple resistance-conferring mutations in the *folA* gene that encodes for DHFR (Fig. 1c)^7,16,18–20^. Antibiotic sensitive *E. coli* populations evolve nearly four orders of magnitude higher TMP resistance by accumulating three to five mutations in *folA*. TMP-resistant *E. coli* populations acquire a promoter mutation (i.e., *c-35t*) that increases DHFR expression, and multiple mutations in the coding region that decreases affinity (increased *K*_i_) of DHFR to TMP molecules^7^. Among all the mutations found in the coding region of DHFR, the most commonly mutated residues were P21, A26, D27, L28, W30, I94, and F153 (Fig. 1d)^7,16^. As TMP is a competitive inhibitor, all of the point mutations in DHFR reduced both TMP and substrate (DHF) affinities simultaneously, with the exception of the L28R mutation^7,21^. Interestingly, unlike other mutations, the L28R mutation reduced TMP affinity while increasing DHF affinity. Also, in our evolution experiments, L28R was the most frequently observed mutation in the coding region of *folA*, with the strongest effect on TMP resistance (Fig. 1d). Moreover, L28R can compensate for the reduced catalytic activity caused by other DHFR mutations such as P21L or W30R^7,22,23^. Our molecular dynamics simulations and biochemical measurements suggested that the L28R mutation leads to a unique and indirect resistance mechanism against TMP: increasing substrate affinity due to newly formed interactions between the mutated enzyme and *p*-aminobenzoyl glutamate tail of the substrate DHF (Fig. 1e and Supplementary Fig. 1)^7,21^. Hence, we concluded that L28R would make an excellent candidate to test our hypothesis of using mutant-specific antibiotic molecules in order to modulate the evolution of antibiotic resistance. Here, we show that an L28R-specific trimethoprim derivative can impede evolution of antibiotic resistance by blocking several evolutionarily viable genetic trajectories.

## Results and discussion

### Structure-guided design of L28R-specific DHFR inhibitor

To gain greater mechanistic insight, we next characterized the atomic structures of wild-type DHFR (DHFR^WT^) and mutant DHFR with an L28R replacement (DHFR^L28R^) using X-ray crystallography. Both DHFR^WT^ (Fig. 1f, resolution 1.9 Å) and DHFR^L28R^ (Fig. 1g, resolution 2.1 Å) were individually co-crystalized with the cofactor NADPH and inhibitor TMP. Distances between the amino acid residues of the enzyme and TMP (Fig. 1h, black values are for DHFR^WT^ and magenta values are for DHFR^L28R^) indicated that there are only subtle differences between these two structures, particularly around the enzyme’s active site. Consequently, we recognized that the 2,4-diamino pyrimidine group of the drug can equally bind to the active site of both DHFR^WT^ and DHFR^L28R^, and should not be altered when designing an L28R-specific DHFR inhibitor. The arginine side chain at residue 28 of DHFR^L28R^ (purple in color, Fig. 1g) is oriented towards the tail of the TMP molecule where the 3,4,5-trimethoxy aryl ring is located. Hence, we predicted that a TMP modification with an appropriate polar group at the 3, 4, or 5 position of this aryl ring might induce additional electrostatic or hydrogen-bonding interactions and thus yield a L28R-specific drug candidate (Fig. 1i).

### Antibacterial activity of L28R-specific DHFR inhibitor

**Fig. 2:**
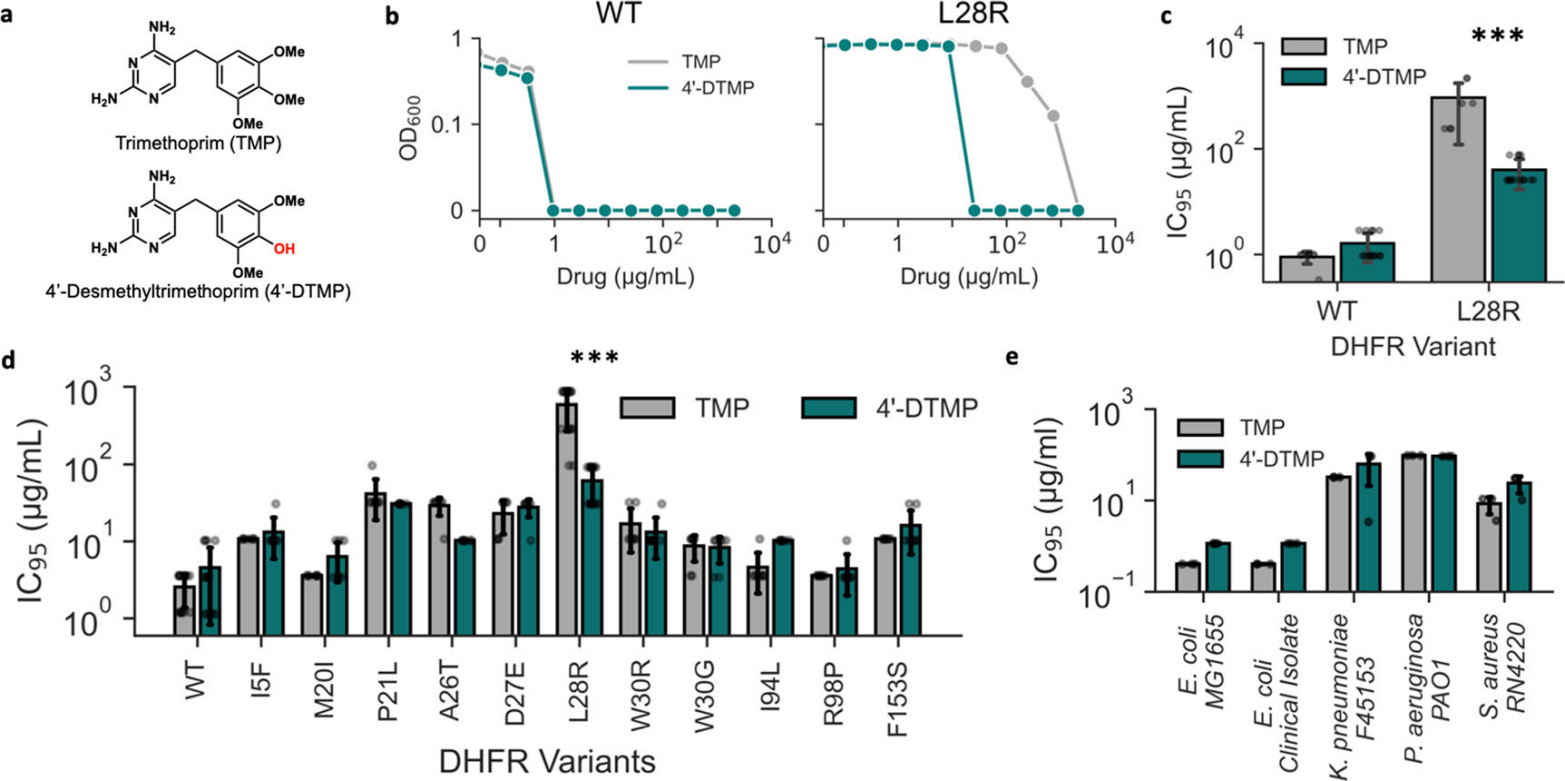
4′-DTMP has enhanced and selective antimicrobial activity against *E. coli* with the L28R mutation in DHFR. **a** Structure of Trimethoprim (TMP) and 4′-desmethyltrimethoprim (4′-DTMP). **b** Representative drug dose response curves for TMP (gray) and 4′-DTMP (teal) against *E. coli* harboring wildtype DHFR (wild-type, left) or DHFR with the L28R mutation (right). All DHFR mutant strains were constructed in MG1655 attTn7::pRNA1-tdCherry (NDL47, see Methods” section) by replacing the chromosomal *folA* gene with the corresponding chemically synthesized *folA* variant sandwiched between kanamycin and chloramphenicol resistance genes. **c** Activity of 4′-DTMP (teal) is indistinguishable from TMP (gray) activity against wildtype *E. coli* (*n* = 7 replicates), whereas 4’-DTMP (teal) has ~30-fold higher antimicrobial activity (*p* = 9.597 × 10^−4^, *n* = 14 replicates) compared to TMP (gray) against *E. coli* with L28R- mutation. **d** Activity of 4′-DTMP (teal) against wild-type and other frequently-observed *E. coli* mutants with single DHFR mutations is indistinguishable from TMP activity (gray) (*n* = 7 replicates), except L28R (*p* = 3.5912 × 10^−6^). **e** Indistinguishable antimicrobial activities of TMP (gray) and 4′-DTMP (teal) against other Gram-negative and Gram-positive bacteria as well as a clinical *E. coli* isolate (*n* = 3 replicates). Student’s *t*-test (two tailed) is used to quantify significance of IC_95_ value differences in all panels (*p < 0.05, **p < 0.01, and ***p < 0.001, error bars show the standard deviation, center of the error bars corresponds to the mean value of the measurements.).

Indeed, we ultimately identified a candidate molecule, 4′-desmethyltrimethoprim (4′-DTMP, Fig. 2a, bottom), which exhibited 30–90-fold enhanced antimicrobial activity against isogenic *E. coli*-L28R compared to native TMP molecule (Fig. 2b, c). Antimicrobial activities of all of the compounds we tested are listed in Supplementary Table 1. Surprisingly, the efficacies of TMP and 4′-DTMP were indistinguishable against wild-type *E. coli* (Fig. 2b, c). Note that unless stated otherwise, all of the *E. coli* strains we used for these measurements are derivatives of the NDL47 strain (MG1655 attTn7::pRNA1-tdCherry). Briefly, we generated a library of *E. coli* with various DHFR mutations by replacing the wild type *folA* gene with the corresponding *folA* variant that is sandwiched between kanamycin and chloramphenicol resistance genes^23^. Next, we tested the efficacies of TMP and 4′-DTMP against *E. coli* strains that harbored one of the 11 point mutations in DHFR known to confer resistance to TMP. We found that 4′-DTMP had increased activity against only the L28R mutation, while exhibiting activity comparable to TMP with respect to other mutations (Fig. 2d). Finally, we tested TMP and 4′-DTMP against a large *E. coli* mutant library containing all possible combinations of P21L, A26T, L28R, W30G, W30R, and I94L mutations in DHFR^7,23^. Overall, 4′-DTMP had better efficacy against most of the mutants containing L28R (Supplementary Table 2 and Supplementary Fig. 2) when compared to TMP. Additionally, we tested the efficacy of 4’-DTMP against other bacterial pathogens: a clinical isolate of *E. coli*, other Gram-negative bacterial species (e.g., *Klebsiella pneumoniae* and *Pseudomonas aeruginosa*), and a Gram-positive *Staphylococcus aureus* strain. Again, we found that 4′-DTMP had similar antimicrobial activities as TMP against both Gram-negative and Gram-positive species (Fig. 2e). When we measured the in vitro affinities (*K*_i_) of TMP and 4′-DTMP to DHFR^WT^ and DHFR^L28R^ using purified DHFR proteins (see “Methods” section) we observed that 4′-DTMP had nearly two-fold higher binding affinity to DHFR^L28R^ compared to TMP at steady state (Supplementary Fig. 3), whereas the *K*_i_ values of TMP and 4′-DTMP were comparable for DHFR^WT^. We also confirmed that, the increased efficacy of 4′-DTMP (compared to TMP) against the L28R mutation is not due to the differences in efflux rates, as the efficacies of TMP and 4′-DTMP were indistinguishable in all *E. coli* strains with other DHFR mutations and impairing the efflux machinery of a drug sensitive *E. coli* strain by deleting the *tolC* gene *(BW25113*:∆*tolC*, Supplementary Fig. 4a, b) did not cause any significant change in the efficacy of 4′-DTMP. Interestingly though, drug accumulation assay using mass spectroscopy with wild-type, L28R, BW25113, and BW25113: ∆*tolC E. coli* indicated that 4′-DTMP accumulated more in L28R *E. coli* compared to other strains after 24 h (Supplementary Fig. 5). These observations all together suggested that the higher accumulation of 4′-DTMP in *E. coli* cells with the L28R mutation was due to stronger interactions between mutated DHFR proteins and 4′-DTMP molecules. However, our attempt to solve the structure of the L28R mutant with 4′-DTMP was not successful.

4′-DTMP was previously reported in the literature but was never used as a mutant specific DHFR inhibitor. It was shown that 4′-DTMP had similar antimicrobial activities as TMP against drug-sensitive *E. coli* bacteria^24^. We characterized toxicity of 4′-DTMP since there was limited information about this molecule in previous literature. In vitro toxicity studies of these two compounds against confluent human cells (ARPE-19) indicated that 4′-DTMP has a similar toxicity profile as TMP up to 1000 μM concentration. However, we found that 4′-DTMP had significant toxicity against dividing cell lines we tested, including ARPE-19, HEK293A, and CHO-DHFR, beyond 500 μM concentration (Supplementary Fig. 6). Although significant, it is not clear whether the relatively higher toxicity of 4′-DTMP will be limiting its potential clinical use as the levels of TMP in plasma and urine samples of patients are generally reported to be around 10 μM^25,26^. Further structural, genetic, and metabolic studies are necessary to gain a deeper understanding of 4′-DTMP’s superiority as a drug against L28R *E. coli*.

### Elimination of L28R mutation from TMP-resistant polyclonal *E. coli* populations

**Fig. 3:**
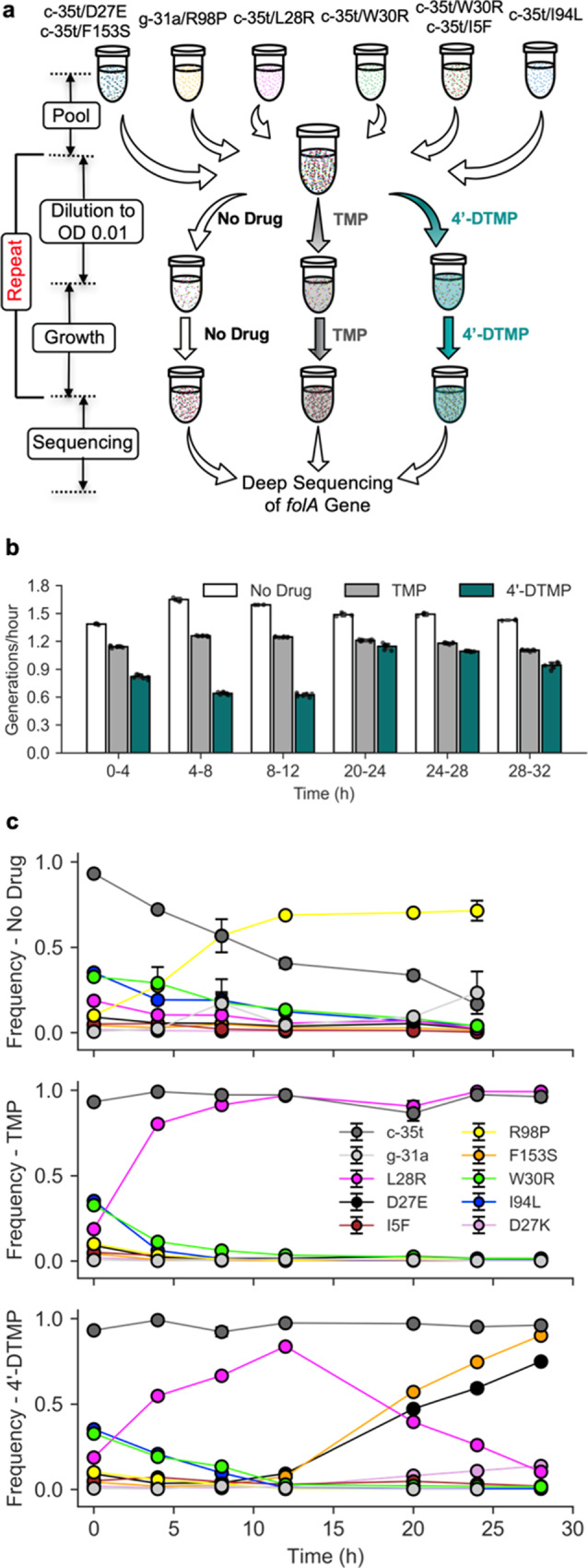
4′-DTMP selectively eliminates mutants carrying the L28R mutation from TMP-resistant populations. **a** Schematic representation of our experiment. A mixture of six bacterial populations with highly TMP-resistant genotypes are propagated for ~32 h, under selection with 500 µM of 4′-DTMP (*n* = 7 replicates), TMP (*n* = 7 replicates), or no drug (*n* = 3 replicates) as a control. DHFR mutations that were dominantly found in these cultures are listed. Cultures were grown for 4 (or 8) hours and they were serially diluted to maintain low cell density and faster growth. Samples were collected at six time points before each dilution event and the *folA* gene of these populations were deep sequenced using MiSeq (please see the SI for details). **b** Average growth rates of cultures in between dilutions are quantified by calculating number of generations per hour (number of generations = log_2_[OD_final_/OD_initial_]). Cultures under 4′-DTMP selection had slowest growth till 12 h and then started to grow faster. Error bars show the standard deviation, center of the error bars corresponds to the mean value of the measurements. **c** Temporal changes in frequencies of DHFR mutations with no drug (top panel), under TMP selection (middle panel), or under 4′-DTMP selection (bottom panel) [Error bars show the standard error on the mean, center of the error bars corresponds to the mean value of the measurements]. The L28R mutation quickly plateaus under TMP selection whereas it was replaced by the D27E and F153S mutations under 4′-DTMP selection. We note that the initial increase in L28R frequency under 4′-DTMP selection is accompanied with slow growth and small population size, further supporting selective activity of 4′-DTMP against the L28R mutation.

We hypothesized that selective elimination of antibiotic resistant subpopulations of bacteria by 4′-DTMP could be an effective strategy to modulate population structures of evolving bacterial cultures. The coexistence of two or more subpopulations of bacteria, also termed as clonal interference, is often observed both in clinical settings as well as laboratory evolution experiments^16,18,27^. Hence, we chose six polyclonal *E. coli* populations that we previously evolved in vitro under TMP selection^7^. These bacterial populations exhibited high levels of TMP resistance, albeit with notable differences in DHFR mutation distribution (Fig. 3a). We first measured resistance of these populations against both TMP and 4′-DTMP (Supplementary Fig. 7). Then we created an even more diverse bacterial population by mixing the six polyclonal cultures in nearly equal ratios (Fig. 3a). We carried out a competition assay by propagating this population in 500 µM of either TMP or 4′-DTMP, as well as in the absence of drug as a control. This process was continued for 32 h using the same concentration of drugs and samples were frozen at six different time points (Fig. 3a). Finally, we calculated fitness changes in mixed populations (Fig. 3b) and quantified frequencies of DHFR mutations by amplicon sequencing of the *folA* gene (Fig. 3c). We found that the L28R mutation quickly increases in frequency and plateaus in the presence of TMP (Fig. 3c, middle) whereas it is eliminated from mixed populations when 4′-DTMP was used (Fig. 3c, bottom). Interestingly, when 4′-DTMP was used, the frequency of the L28R initially increased but was later outcompeted by D27E and F153S mutations. The time point at which the L28R mutation started to decrease in abundance coincided with an almost two-fold increase in the growth rate of the populations (Fig. 3b). Additionally, until this tipping point was reached, the cell density of bacterial populations growing with 4′-DTMP were always low, suggesting poor fitness of mixed populations in the presence of 4′-DTMP. Regardless of the drug used, frequencies of the promoter mutations were always constant indicating that DHFR overexpression is an effective resistance mechanism for both TMP and 4′-DTMP. Lastly, the R98P mutation gradually swept all other mutations including the promoter mutation when no drug was used (Fig. 3c, top), implying its relative fitness advantage over other mutations in the absence of drug selection.

### 4′-DTMP impedes evolution of antibiotic resistance

**Fig. 4:**
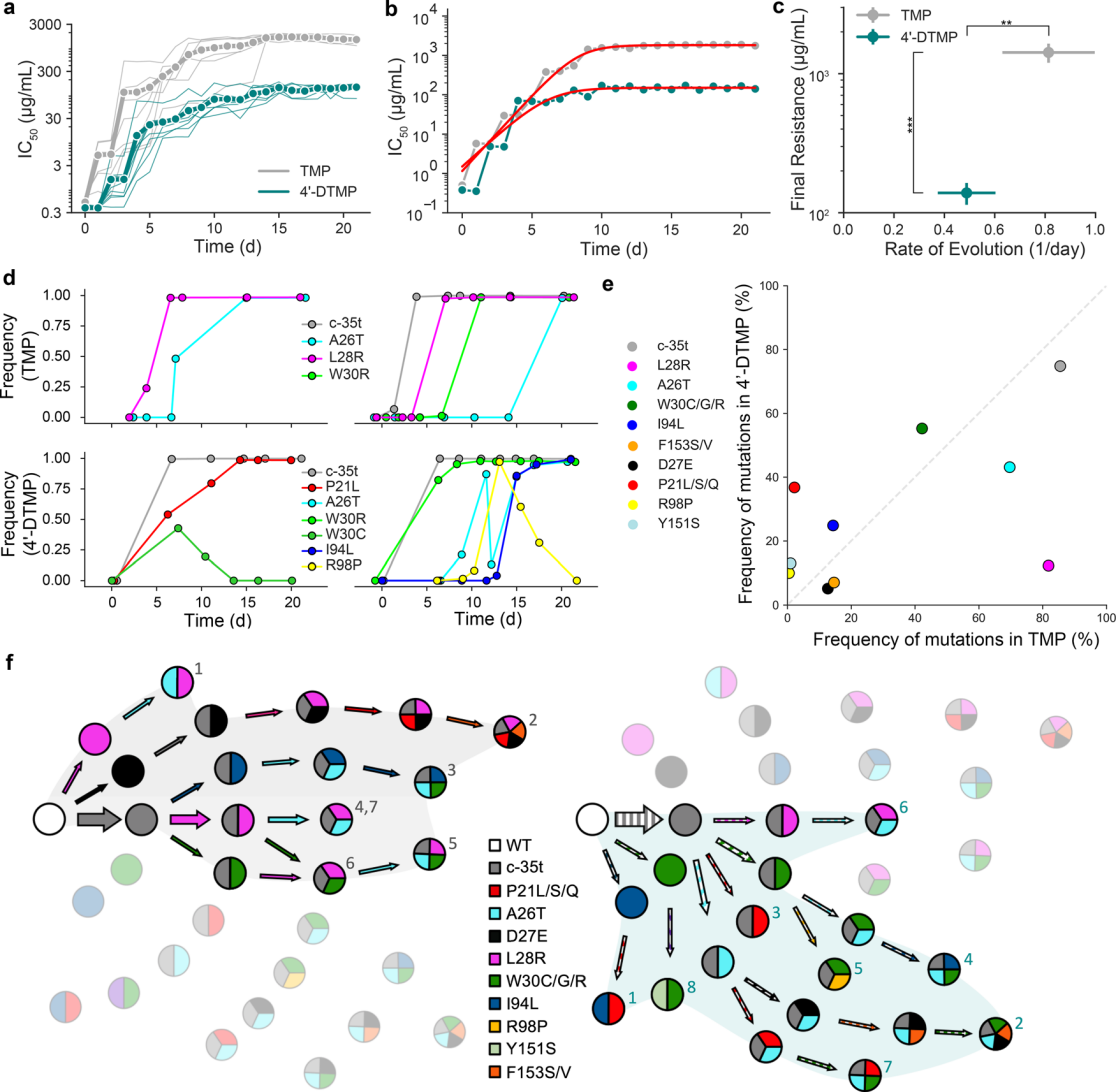
L28R-specific 4′-DTMP slows down evolution of antibiotic resistance in the morbidostat. **a** Overlaid changes in resistance levels (IC_50_) over time for *E. coli* populations evolved in parallel under inhibition by 4′-DTMP (teal, *n* = 8 replicates) and TMP (gray, *n* = 7 replicates), demonstrating an increase in resistance by ~100-fold and ~2000-fold, respectively. Day 0 corresponds to parent strain. Light lines represent individual cultures; dark lines with filled circles represent mean values. **b** A log-logistic function was used to estimate final resistance levels and rate of evolution for evolved *E. coli* cultures. Representative example showing log-logistic function fits in red lines. **c** *E. coli* cultures acquired lower resistance levels (*p* = 2.10e^−8^) at slower rates (*p* = 0.0016) when evolved in 4′-DTMP compared to cultures evolved in TMP. Student’s *t*-test (two-tailed) is used to quantify significance of differences in all panels (*p < 0.05, **p < 0.01, and ***p < 0.001, error bars show the standard deviation, center of the error bars corresponds to the mean value of the measurements). **d**, Representative examples of time-resolved changes in DHFR as measured by deep sequencing of the *folA* gene throughout evolution in the morbidostat (top row for TMP- and bottom row for 4′-DTMP-evolved cultures). *E. coli* cultures develop TMP resistance by acquiring the L28R mutation (6 out of 7 cultures) whereas *E. coli* cultures become 4′-DTMP resistant without acquiring L28R (only 1 out of 8 cultures acquired L28R). **e** Final frequencies of observed resistance-conferring DHFR mutations under TMP and 4′-DTMP selection on day 21 were compared. The frequency of L28R mutation (magenta) was drastically reduced from ~80% (in TMP-evolved group) to less than 15% (4′-DTMP-evolved group). **f** Observed evolutionary trajectories leading to resistance in *E. coli*. The observed DHFR mutations are indicated by circles, filled with colors. The solid arrows represent the mutations acquired by *E. coli* evolved under TMP selection (left, gray background) whereas the dashed arrows represent the mutations acquired by *E. coli* evolved under 4′-DTMP selection (right, teal background). The color of the arrows indicate the specific mutations observed. The width of the arrow corresponds to the observed frequency of that mutation. The number on a circle indicates the culture number (gray for TMP and teal for 4′-DTMP evolved cultures, **f**).

In order to better understand the long-term effects of TMP and 4′-DTMP use on the evolution of *E. coli* antibiotic resistance, we evolved an initially isogenic, antibiotic sensitive *E. coli* strain (TB194, MG1655: attP21-PR-mCherry-chlorR) for 21 days (~10–15 generations per day) using TMP (Fig. 4a, gray lines, *n* = 7 replicates) and 4′-DTMP (Fig. 4a, teal lines, *n* = 8 replicates) in a morbidostat. All of the populations evolved under TMP selection rapidly acquired very high levels of resistance and their MIC values were beyond the maximum solubility of TMP in growth media (~2.5 mg/mL) we used in this study. However, bacterial populations evolving under 4′-DTMP selection acquired resistance at a slower pace than the populations evolved under TMP selection (Fig. 4a–c and Supplementary Fig. 8). Furthermore, the final resistance levels of the populations evolved in 4′-DTMP were ~10 fold lower than those evolved in TMP (Fig. 4c). Bacterial populations evolved under TMP selection were also resistant to 4′-DTMP (Supplementary Fig. 9). Similarly, bacterial populations evolved under 4′-DTMP selection were found to be highly resistant to TMP (Supplementary Fig. 9). Therefore, both TMP and 4′-DTMP select for cross-resistance to one another. However, bacterial populations evolved under 4′-DTMP selection grow slower and have significantly longer doubling times compared to the populations evolved under TMP (Supplementary Table 3, 63.7 ± 12.5 min and 49.8 ± 10 min, respectively; Student’s *t*-test, *p* = 0.04). In summary, long-term evolution of *E. coli* under TMP and 4′-DTMP selection revealed that 4′-DTMP impedes the evolution of antibiotic resistance.

Analysis of genetic changes in *E. coli* DHFR during the evolution of resistance experiments revealed that 4′-DTMP successfully blocks evolutionary trajectories leading to L28R mutation (Fig. 4). By selecting numerous samples archived from bacterial cultures evolved in the morbidostat, we observed that the populations sequentially accumulated several mutations in *folA* gene (as determined by amplicon sequencing and quantification of temporal changes of DHFR mutations; see “Methods” section)^28^. For both TMP and 4′-DTMP, we observed that the populations sequentially accumulated several mutations, and clonal interference and sweeping events were frequently observed (Fig. 4d). In both the cases, the majority of the populations acquired a promoter mutation (*c-35t*) early in their evolution (Fig. 4d–f). Strikingly, only one out of eight populations evolving under 4′-DTMP acquired the L28R mutations, whereas L28R was commonly observed in six out of seven populations when evolved under TMP selection (Fig. 4d–f, *p* = 0.017, Wilcoxon). We note that L28R mutation was observed in ~70% of our previous morbidostat experiments (*n* = 33) and experiments done by others^7,16,18,29^. The difference in final frequencies of the observed mutations under TMP and 4′-DTMP selection at the end of 21 days are displayed in Fig. 4e. The frequency of L28R mutation was drastically reduced from ~80 to ~15%. As summarized in Supplementary Table 3, populations evolved under 4′-DTMP selection also grow slower compared to populations evolved under TMP selection, further supporting the critical evolutionary role of the L28R mutation in maintaining the catalytic activity of DHFR. As summarized in Fig. 4f, elimination of L28R, the most beneficial DHFR mutation under TMP selection (Fig. 4f, left, trajectories highlighted with gray background), impedes the evolution of antibiotic resistance by diverting genetic trajectories (Fig. 4f, right, trajectories highlighted with teal background). Using 4′-DTMP instead of TMP, therefore, eliminates genetic trajectories leading to genotypes with the L28R mutation. In these genetic trajectories, we do not observe a dominant mutation that replaces L28R. Instead, we observe several DHFR mutations with slightly elevated frequencies compared to mutations observed in the presence of TMP (Fig. 4e).

By comprehensively understanding the molecular evolution of TMP resistance in *E. coli*, we identified and targeted a common mutation (L28R with 4′-DTMP) and subsequently effectively slowed down evolution of antibiotic resistance. Use of 4′-DTMP eliminates genetic trajectories that accumulate the L28R mutation, the most beneficial resistance-conferring DHFR mutation against TMP. The L28R mutation has positive epistatic interactions with other resistance-conferring DHFR mutations, as L28R compensates for catalytic deficiencies caused by these mutations^7,16,21–23^. Therefore, blocking the L28R mutation diverts evolutionary trajectories towards genotypes with different DHFR mutations. Whether the use of 4′-DTMP will also slow down evolution of antibiotic resistance in clinical strains of *E. coli* or other pathogenic bacterial species is currently unknown to us, and addressing this question requires further laboratory evolution experiments and other experiments in animal models. Our study is an important demonstration of how information from laboratory evolution experiments and structural analysis of resistance-conferring mutations could guide efforts for developing novel antibiotic molecules and improving existing antibiotics. Furthermore, for drug targets known to exhibit evolutionary plasticity (e.g., DHFR), close monitoring of bacterial evolution and developing mutant-specific antibiotic molecules may not only eliminate resistant bacteria, but may also increase long term efficacy of antibiotic therapies by blocking evolutionary trajectories that lead to resistance genotypes.

## Methods

### Data collection, structure determination and refinement, final model validation, and uncertainty

X-ray diffraction data sets for both crystals were collected at the Advanced Photon Source (APS), Structural Biology Center at Sector 19-ID. HKL3000 was used to process both wild-type and L28R data sets^30^. Computational corrections for absorption in a crystal and imprecise calculations of the Lorentz factor resulting from a minor misalignment of the goniostat were applied^31,32^. Anisotropic diffraction was corrected to adjust the error model and to compensate for a radiation-induced increase of non-isomorphism within the crystal^33–35^. For the wild-type crystal, data were merged to 1.9 Å resolution, but diffraction was highly anisotropic with diffraction in the *a* and *b* directions reaching only ~2.2 Å (<I>/<σ(I)> ~2 at 2.25 Å, and ~0.1 at 1.9 Å), with the *c* direction extending beyond 1.9 Å (<I>/<σ(I)> ~37 at 2.25 Å and ~7 at 1.9 Å). Such strong anisotropy affects all steps of data analysis and we had to consider its impact during the structure solution and refinement. Indexing, integration, and scaling indicated P3_x_21 symmetry. The space group ambiguity was resolved during molecular replacement, when the space group was determined to be P3_2_21. The L28R mutant crystals grew in the same symmetry (P3_2_21) and their diffraction displayed strong anisotropy as well. Data were merged to 2.1 Å resolution, with diffraction in the *a* and *b* directions reaching only ~2.2 Å (<I>/<σ(I)> ~1.9 at 2.65 Å, and ~0.06 at 2.1 Å), with the *c* direction extending beyond 2.1 Å (<I>/<σ(I)> ~45 at 2.65 Å and ~8.6 at 2.1 Å). The data processing statistics are presented in Table 1.

**Table 1:** Data processing and refinement statistics for 6XG5 and 6XG4.

	Wild-type (6XG5)	L28R (6XG4)
*Data collection*^a^		
Space group	P3_2_21	P3_2_21
*Cell dimensions*		
*a*, *b*, *c* (Å)	*a* = *b* = 61.81, *c* = 104.63	*a* = *b* = 61.55, *c* = 104.66
*α*, *β*, *γ*(°)	*α* = *β* = 90, *γ* = 120	*α* = *β* = 90, *γ* = 120
Resolution (Å)^b^	50.00–1.90 (1.93–1.90)	50.00–2.10 (2.12–2.10)
║ a, b	2.25	2.65
║ *c*	1.90	2.10
*R*_pim_^c^	0.026 (NA)	0.025 (NA)
*R*_meas_	0.058 (NA)	0.076 (NA)
CC1/2	0.982 (0.552)	1.000 (0.672)
*<I*>/<σ*I*>	33.4 (0.9)	37.5 (0.7)
Completeness (%)	99.2 (87.3)	99.9 (100.0)
Multiplicity of observations	6.1 (4.3)	9.3 (8.8)
Number of reflections	18,711 (790)	13,880 (334)
*Refinement*^a^		
Resolution (Å)	47.70–1.90 (1.95–1.90)	37.37–2.10 (2.16–2.10)
No. reflections	14,620 (339)	9837 (212)
*R*_work_/*R*_complete_^d^	23.62/26.24	24.9/27.8
*No. atoms*		
Protein	1333	1297
Water molecules	135	131
Others	76	75
*Average B-factors* (Å^2^)		
Protein	31.2	38.4
Ligands	21.4	27.1
Ions	48.5	59.6
Water molecules	29.3	31.7
*R.m.s deviations*		
Bond lengths (Å)	0.006	0.006
Bond angles (°)	1.385	1.417
Completeness (%)^e^	77.7 (25.0)	71.0 (21.7)
Molprobity score	1.83 (80th percentile)	2.45 (45th percentile)
Clashscore RCSB code	7.65 (89th percentile)	8.95 (90th percentile)

^a^Values in the parentheses represent the last resolution shell. Please note that data sets were processed and refined with different shell divisions due to automatic procedures splitting data sets so that about equal numbers of reflections are present in each shell.

^b^The resolution is reported in two different directions because of the strong anisotropy.

^c^The high crystallographic symmetry and high redundancy result in the values of *R*_pim_ and *R*_merge_ exceeding 1.000. Such values do not have statistical meaning; therefore, we report them as NA.

^d^Please note that we used complete cross validation as described in the text.

^e^Please note that the nominal data completeness is very high i.e., we collected and processed complete data sets. However, we used elliptical truncation before refinement and that kept only ~25% of reflections at high resolution.

The literature and RCSB searches revealed that the *Escherichia coli* DHFR crystal form we obtained was observed before (PDB codes: 1drh, 7dfr, 1dre, 1rh3)^36–38^, including the complex of wild-type with TMP. However, the complex of DHFR with TMP was never deposited, very likely due to severe refinement difficulties that such high anisotropy would cause with a lower resolution dataset (the complex was reported by Sawaya and Kraut^36^).

The wild-type structure was solved with the molecular replacement method, using MOLREP^39^ with 1RX2.pdb as a search model. The top solution (CC = 0.674) was refined with REFMAC^40,41^. The wild-type model was used to perform isomorphous replacement with the L28R dataset using MOLREP^39^ and then rebuilt and refined with REFMAC. The final rounds of refinement for both structural models were performed by iteratively applying REFMAC refinement and manual rebuilding and corrections in COOT^42,43^. We also used TLS restraints in refinement^44^ with the TLSMD server providing the best combination of TLS bodies^45^. The results of refinement are summarized in Table 1. The model quality was validated with Molprobity^46,47^ and assessed to be satisfactory on the basis of the data resolution with Molprobity scores of 1.74 (wild-type) and 2.28 (L28R), which correspond to the 86th percentile for wild-type in comparison with the set of 12,147 PDB deposits solved at resolutions from 1.90 Å ± 0.25 Å and to the 60th percentile for L28R in comparison with the set of 11,758 PDB deposits solved at resolutions 2.10 Å ± 0.25 Å.

During refinement, we noticed that our R-free dataset that consisted of 5% of all reflections is too limited both for the wild-type and the mutant to assure stability of R-free during refinement. It contained only 763 reflections for wild-type and 512 reflections for the mutant. The problem of having too insufficient a dataset to assure a good cross-validation is well-recognized, but usually it is solved by increasing the number of reflections used for R-free calculations^48,49^. However, we were concerned that excluding a higher percentage of reflections, in combination with anisotropic diffraction and limited completeness at higher resolution due to elliptical truncation, would deteriorate the quality of the electron density maps, and consequently would limit our ability to model the DHFR structure and water molecules coordinated by the macromolecular chain. Therefore, we performed complete cross-validation^48^ for both datasets, with the R-free flags transferred between the wild-type and L28R structures. In the CCP4 package^50,51^, the R-free flag is assigned by the program FREEFLAG during the initial transformation of diffraction data intensity to structure factor amplitudes. We selected 5% of the reflections as the R-free set and therefore the FREEFLAG program generated 20 subsets of reflections that can be used for R-free calculations, each associated with a flag ranging from 0 to 19. By default, R-free is calculated in REFMAC with reflections having flag = 0, but one can select any other flag if desired. To achieve full cross validation, in the last cycle of refinement before the deposition, we run the final model that was refined with the R-free flag 0, through 21 identical refinements, 20 using flags 0–19 to calculate R-free and one using all reflections so that we could perform final examination of the electron density maps. Subsequently, we averaged arithmetically 20 R-free values obtained from these refinements and these values are reported in the PDB as R and R-free factors. An analogous procedure was proposed and extensively tested by Luebben and Gruene^52^. The complete cross validation resulted in R and R-free values expected for anisotropic data having limited completeness in the highest resolution shells, while refinements against the individual R-free sets showed discrepancies expected in this situation^48^.

The structural models for the wild-type and L28R were deposited to RCSB^53,54^ under codes 6XG5 and 6XG4, while the diffraction datasets associated with them have been deposited with the Integrated Resource for Reproducibility in Macromolecular Crystallography at www.proteindiffraction.org^55^. Figure 1f, g were generated using PyMol.

### Bacterial strains

#### Wild-type *E. coli*

The wild-type *E. coli* strain we used for the measurements is a derivatives of the NDL47 strain (MG1655 attTn7::pRNA1-tdCherry, a gift from Johan Paulsson Lab at Harvard Medical School) that has the wild-type DHFR (*folA*) gene sandwiched between the kanamycin and chloramphenicol resistance cassettes. We used the chromosomal integration method described by Datsenko and Wanner (PNAS, 2000) for generating the DHFR mutation library.

#### DHFR mutant *E. coli*

We generated a library of *E. coli* with various DHFR mutations by replacing the wild type *folA* gene of the NDL47 strain with the corresponding *folA* variant that is sandwiched between kanamycin and chloramphenicol resistance genes (Palmer et al.^23^).

### Protein overexpression and purification

The L28R mutation in *folA* gene was constructed by using Quick-Change Site-Directed Mutagenesis kit (Stratagene). 6×HisTag was added at C-termini of the protein sequence for wild-type and L28R constructs. The constructs were cloned into the expression plasmids pET24a-KanR. BL21 *E. coli* cells were transformed with pET24a-*folA*−6×HisTag and were grown overnight in selective media (LB + Kan) and then diluted 100 times into TB media for further growth at 30 °C. Protein overexpression was induced when OD_600_ reached 0.6–0.8, with 1 mL of 250 mM IPTG per 1 L of the medium, and the temperature was decreased to 18 °C for further growth, with 220 rpm shaking. Recombinant protein was purified using Ni-NTA columns (Qiagen), and dialyzed overnight against 50 mM Tris-Base, pH 8.0, 0.5 M NaCl.

### Crystallization of wild-type and L28R DHFR proteins

Both wild-type and L28R variant of DHFR proteins at 9 mg/mL concentration were mixed with 10 mM NADPH and 2 mM TMP and incubated overnight at 4 °C. Precipitated material was removed by centrifugation at 12,000×*g* for 5 min. The TMP complex was crystalized by hanging drop vapor diffusion, mixing the protein 1:1 with a reservoir solution containing 0.1 M sodium citrate tribasic dihydrate (pH 5.6), 0.15 M ammonium acetate and 17.5 or 20% PEG4000 for DHFR^WT^ and DHFR^L28R^, respectively. DHFR^L28R^ was incubated at 16 °C whereas wild type was incubated at 20 °C, and crystals were formed in 1–2 weeks. Crystals were cryo-protected in a mother liquor solution containing 25–30% of glycerol, and cryo-cooled in liquid nitrogen.

### Preparation of 4′-DTMP

Please see Supplementary Note 1 for the synthesis and characterization of 4′-DTMP.

### Drug solutions

Freshly prepared drug solution of TMP and 4′-DTMP was used in every experiment. A 200 mM stock solution in DMSO was prepared, which was further diluted to required concentration using M9 minimal media supplemented with 0.4% glucose (Fisher Scientific B152-1), 0.2% amicase (MP Biomedicals 104778), 2 mM MgSO_4_ (Fisher Scientific M63-500), 100 µM of CaCl_2_ (Fisher Scientific S25222A) and filtered. They were kept at room temperature in glass bottles wrapped with aluminum foil to avoid light induced drug degradation.

### IC_95_ measurements

Bacterial cultures were grown at 37 °C in M9 minimal medium supplemented with 0.4% glucose (Fisher Scientific B152-1), 0.2% amicase (MP Biomedicals 104778), 2 mM MgSO_4_ (Fisher Scientific M63-500), and 100 µM of CaCl_2_ (Fisher Scientific S25222A). Overnight grown cultures were diluted to optical density (OD) of 0.001 using drug solution in M9 media. Plates were incubated in 37 °C with continuous shaking in Liconic Shaking Incubator and growth is measured with Tecan Plate Reader Infinite M200. Background optical density levels are subtracted from all wells. IC_95_ was defined as minimum drug concentration at which the bacterial growth is inhibited by 95% compared to growth in the absence of drugs (Fig. 2c–e, Supplementary Tables 1,  2, Supplementary Figs. 2,  4,  7, and  9).

### Steady-state kinetic measurements

Reactants of DHFR reaction [DHF (Sigma-Aldrich D7006) and NADPH (Sigma-Aldrich N7505)] has absorbance at 340 nm where the products (THF and NADP^+^) do not absorb light. Concentrations of DHF and NADPH were measured using extinction coefficients of 6200 M^−1^ cm^−1^ at 340 nm and 28,000 M^−1^ cm^−1^ at 282 nm. Using UV/Vis Spectrophotometer (Perkin Elmer LAMBDA 650), we measured reaction progression with 1 s time intervals with two measurement cells. First cell was the sample cuvette containing the reaction components (DHFR, DHF, and NADPH) and the second was the reference cell containing only NADPH and DHFR in it. Biochemical measurements were done at 25 °C in MTEN buffer (pH ~ 7) which includes, 50 mM MES hydrate (Sigma-Aldrich M8250), 25 mM Tris–Base (Fisher Scientific B152-1), 25 mM ethanolamine hydrochloride (Sigma-Aldrich E6133), 100 mM NaCl (Fisher Scientific S271-3), and 5 mM DTT (Fisher Scientific BP172-25) which is added fresh before starting the reaction. MTEN solution containing 300 nM DHFR protein and 200 mM NADPH was prepared. To this 12.5 mM DHF and 200 mM NADPH solution was added preceding the data collection. Data collection was stopped when all the DHF was consumed which happens when the curve reached a plateau down below zero. The process was repeated using 12 different concentrations of TMP or 4′-DTMP ranging from 0.1 nM to 100 μM. A custom MatLab code was used to calculate the *K*_i_ values (Supplementary Fig. 3).

### Intracellular drug concentration measurement

Wild-type, L28R, BW25113, and BW25113:∆TolC *E. coli* strains were incubated overnight at 37 °C in M9 media. Two hundred micromolar DMSO stock was prepared for the drugs TMP and 4′-DTMP. The drug solution was diluted with M9 media to adjust the concentration of drug to 6 μM. 2.5 mL of drug solution was added to a 15 mL conical tube [two time points (1 and 24 h) and three replicates for each set of conditions]. OD was calculated for the *E. coli* strains. Required volume (~500 µL) of *E. coli* cells were added to the conical tube such that at *t* = 0, concentration of the drug is 5 µM and the starting OD value is ~0.30 [1 OD = 5 × 10^8^ cells]. The cultures were incubated at 37 °C. After 1 h, OD values of one set of cultures were recorded, the cultures were transferred to 5 mL centrifuge vials and centrifuged at 4680 rpm for 3 min. The supernatant was collected and stored at 4 °C. The pellets were re-suspended in 200 µL of ice-cold water, centrifuged at 4680 rpm for 3 min. The pellets are collected and flash-frozen using liquid nitrogen. The other set of cultures were incubated for 24 h. After 24 h, OD values of the cultures were recorded and same steps were followed. The pellets and supernatants were stored for the Mass-spec analysis.

### Processing

#### Supernatant

Two hundred and fifty microliter of blank (M9 media) or supernatant was aliquoted into Eppendorf tubes. Blanks were spiked with varying concentrations of each drug to create a standard curve. Each sample was mixed with 0.5 mL of methanol crash containing 0.15% formic acid and 45 ng/mL *n*-benzylbenzamide internal standard (final 0.1% formic acid and 30 ng/mL IS). The mixture was vortexed for 15 s, incubated 10 min at RT and then centrifuged twice at 16,100×*g* at 4 °C. The supernatant was analyzed by LC-MS/MS.

#### Cell pellets

The cell pellets were re-suspended in M9 media. After the resuspension, each sample possessed the same OD value. Two hundred and fifty microliter of blank (M9 media) or the re-suspended cell pellet was aliquoted into Eppendorf tubes. Blanks were spiked with varying concentrations of each drug to create a standard curve. Each sample was mixed with 0.5 mL of methanol crash containing 0.15% formic acid and 45 ng/mL *n*-benzylbenzamide internal standard (final 0.1% formic acid and 30 ng/mL IS). The mixture was vortexed for 15 s, incubated 10 min at RT and then centrifuged twice at 16,100×*g* at 4 °C. The supernatant was analyzed by LC-MS/MS and the resulting precipitated material saved for quantitation of protein for normalization of each sample.

The precipitated protein resulting from organic extraction of cell pellets was re-suspended in 250 µL of 0.1 M NaOH, boiled for 5 min, and 5 µL was mixed with 200 µL of 1:50 B:A reagent (Thermofisher BCA Kit). A BSA standard curve was prepared in H_2_0 and mixed in the same ratio. The samples were incubated 30 min at 37 °C and read at 562 nM.

### Analytical LC-MS/MS methods

Supernatants from experiments evaluating intracellular levels of TMP or 4′-DTMP were evaluated by LC-MS/MS using an AB Sciex (Framingham, MA) 4000 QTRAP^®^ mass spectrometer coupled to a Shimadzu (Columbia, MD) Prominence LC to determine the amount of compound present. Standard curves were generated using blank M9 media spiked with known concentrations of compound and processed as described above. Analytes were detected with the mass spectrometer in positive MRM (multiple reaction monitoring) mode by following the precursor to fragment ion transition for two daughter ions. Only one parent daughter pair, indicated here, was used for quantitation for each analyte: TMP 291.092 to 230.0; 4′-DTMP 277.169 to 261.200. The transition 212.1 to 91.1 was monitored for the internal standard, *n*-benzylbenzamide. An Agilent C18 XDB column (5 micron, 50 × 4.6 mm) was used for chromatography with the following conditions: Buffer A: dH20 + 0.1% formic acid, Buffer B: methanol + 0.1% formic acid; flow rate 1.5 mL/min for gradient conditions: 0–1.0 min 3% B, 1.0–2.0 min gradient to 100% B, 2.0–3.5 min 100% B, 3.5–3.6 min gradient to 3% B, 3.6–4.5 3% B. The concentrations of drug in each time-point sample were quantified using Analyst software (Sciex). A value of 3-fold above the signal obtained from blank plasma was designated the limit of detection (LOD). The limit of quantitation (LOQ) was defined as the lowest concentration at which back calculation yielded a concentration within 20% of theoretical. The data were plotted using Prism (Supplementary Fig. 5).

Note: No interconversion of TMP to 4′-DTMP or vice-versa was detected in these experiments.

### Toxicity assay

To test for potential cytotoxicity, ARPE-19 cells (CRL-2302, STR verified, University of Arizona Genetics Core), CHO-DHFR (CRL-9096, ATCC, Manassas, VA) or HEK293A (R70507, Life Technologies, Carlsbad, CA) cells were plated in black, clear-bottom 96-well plates (Corning, Corning, NY) at a density of 5000 and 10,000 cells/wells, respectively, and allowed to attach overnight. The next day, cells were treated with DMSO, TMP, or 4′-DTMP (31.25–2000 μM, final concentration, in triplicate) for 24 h. Media was removed and replaced with 50 μL/well of room temperature (RT) Cell Titer Glo 2.0 (Promega, Madison, WI) diluted 1:1 in Hanks buffered salt solution (Sigma Aldrich). Plates were mixed at RT for 5 min on an orbital shaker, incubated at RT for an additional 5 min, and luminescence was read on a Synergy 2 plate reader (10 s integration/well, BioTek, Winooski, VT). Results shown are representative of three independent experiments (Supplementary Fig. 6).

### Propagation of mixed TMP-resistant populations

From our previous evolution experiments, six TMP-resistant bacterial populations with c-35t/D27E/F153S, g-31a/R98P, c-35t/L28R, c-35t/W30R, c-35t/W30R/I5F, and c-35t/I94L mutations were chosen. These mutations were found by Sanger sequencing of randomly selected colonies isolated from populations. The individual minimum inhibition concentration (MIC) values of these populations were determined (Supplementary Fig. 7). From the drug dose response curves, a sub-inhibitory drug concentration of 500 µM was chosen such that the sweeping events would be observed in a large time window. All six populations were individually grown overnight, adjusted to the same OD_600_ value and then pooled in equal volume so that their initial frequencies in the pool would be nearly equal. The pool was then divided into three groups of tubes and diluted to 0.01 OD_600_ with minimal M9 media (20 mL) containing 500 µM of drug (three replicates with no drug, seven replicates with TMP, and seven replicates with 4′-DTMP, Fig. 3a). These cultures were grown at 37 °C for 4–8 h so that they would normally be growing at an exponential rate if no antibiotics were added. At the end of each growth cycle, cell densities were recorded for all cultures, a small fraction of the populations were frozen for sequencing and the remaining cultures were further diluted to 0.01 OD_600_ with fresh media (20 mL) containing 500 µM of drug. This process was repeated till 32 h using same concentration of drugs and the samples were frozen at six different time points. Finally, we calculated fitness changes in mixed populations (Fig. 3b) and quantified frequencies of DHFR mutations by amplicon sequencing of the *folA* gene (Fig. 3c).

### Long-term evolution of *E. coli* under TMP or 4’-DTMP selection using morbidostat

We started morbidostat experiments with wild type (drug sensitive) isogenic *E. Coli* cells (TB194: attP21-PR-mCherry-chlorR, a gift from Tobias Bergmiller lab) that were frozen at –80 °C. We thawed and diluted the cells 1:1000 in M9 media and transferred ~15 mL of the solution into autoclaved morbidostat culture tubes. We assigned seven tubes for TMP and eight tubes for 4′-DTMP. The optical density of the starting culture was calculated after subtracting the average voltage value within the first 30 s. We did not make any injections of drug solutions or fresh media into the culture until the OD of the culture exceeds 0.015 in order to allow cells to adapt their environment. This waiting time is usually around 2 h. After this waiting time, there are injections of fresh media or drug solutions every 18 min. Each injection takes 1 min and is followed by 17 min of growth cycle. Waste pump periodically runs during growth cycle but is turned off when injections are made into the culture to avoid suction of the injected liquid. The total volume inside the culture tube is ~15 mL and injection pumps are operated at ~2.16 mL/min flow rate. Each injection generates ~12.6% dilution of the growing cultures. After every growth cycle, we calculated the growth rate of the cell population, initial and final OD values during the growth cycle, and the drug concentration. The cultures were transferred to a new set of tubes every day, to reduce the chances of forming biofilms. The experiment was continued for 21 days and the drug concentrations used were gradually increased by 5-folds as the populations were acquiring resistance. Samples from each tube were frozen every day. After 21 days, IC_50_ values of TMP and 4′-DTMP were calculated for all the samples and plotted the fitness changes against time (Fig. 4a–c and Supplementary Fig. 8). Finally, we quantified the frequencies of DHFR mutations in those populations by deep amplicon sequencing of the *folA* gene (Fig. 4d–f).

### Deep sequencing of *folA* gene using MiSeq

The frozen glycerol stocks of the bacterial populations were warmed to RT, 25 µL of each population were individually diluted to 1.0 mL using M9 media and grown overnight at 37 °C, to make sure that all the populations have similar OD values. A lysis buffer was prepared by adding 10 µL of Triton X-100 (Sigma Aldrich, T8787) to 10 mL of Tris EDTA buffer. Ten microliter of overnight culture was mixed with 30 µL of lysis buffer, heated at 95 °C for 5 min, and centrifuged at 13,000×*g* for 10 min. The supernatant was used as DNA template in PCR. The *folA* gene was amplified in two portions using the primers listed in Supplementary Table 4.

PCR mixture (15 µL total volume) was prepared by mixing 3.0 µL of DNA template, 0.75 µL of each forward and reverse primer, 7.5 µL of Q5® High-Fidelity 2× Master Mix (BioLabs) and 3.0 µL of H_2_O. Thermocycler was set to 98 °C for 3 min, [98 °C for 15 s, 56 °C for 30 s, 72 °C for 1 min] × 25 cycles, 72 °C for 5 min then 4 °C. All the PCR products were purified using Nucleospin® Gel purification kit (Macherey-Nagel). Library preparation for MiSeq was done using Illumina MiSeq 2 × 250 bp kit, following the standard protocol provided by the vendor. Concentrations of the amplicons were measured using Qubit® assay. Sequencing was done in house using an Illumina MiSeq instrument. Results were analyzed and plotted using custom Python scripts (Figs. 1d,  3c, and  4d–f).

### Reporting summary

Further information on research design is available in the Nature Research Reporting Summary linked to this article.

## Supplementary information

Supplementary Information

Reporting Summary

## Data Availability

Data supporting the findings of this study are available within the paper and its Supplementary Information. The X-Ray crystal structures have been deposited to PDB with 10.2210/pdb6XG4/pdb (L28R) and 10.2210/pdb6XG5/pdb (wild-type). Corresponding raw data for each figure and table used in the manuscript and the supplementary files are accessible free of cost from GitHub (https://github.com/erdaltoprak-zz/NatureCommunication2021_Manna.git). The raw sequencing data are deposited to NCBI with accession code PRJNA717019. Link to the source data files are listed in the “Source Data” file provided with this paper. Source data are provided with this paper.

## Source data

Source Data

## Code availability

All the codes used to plot the figures of this manuscript and the supplementary information of this paper are available from Zenodo (10.5281/zenodo.4630929).
