# Supplementary material for: A trimethoprim derivative impedes antibiotic resistance evolution
Source: Nat Commun. 2021 May 19;12:2949. doi: 10.1038/s41467-021-23191-z (PMC8134463; doi:10.1038/s41467-021-23191-z)
Supplement: Supplementary file 1 — Supplementary Information [file 41467_2021_23191_MOESM1_ESM.pdf]

A trimethoprim derivative impedes antibiotic resistance evolution

Madhu Sudan Manna[1,2], Yusuf Talha Tamer[1], Ilona Gaszek[1], Nicole Poulides[1], Ayesha Ahmed[1], Xiaoyu Wang[2], Furkan C.R. Toprak[3], DaNae Woodard[4], Andrew Y. Koh[5,6], Noelle S. Williams[2], Dominika Borek[7], Ali Rana Atilgan[8], John D. Hulleman[4,9], Canan Atilgan[8], Uttam Tambar[2] & Erdal Toprak[1,9,*]

[1] Green Center for Systems Biology, University of Texas Southwestern Medical Center, Dallas, Texas, USA;

[2] Department of Biochemistry, University of Texas Southwestern Medical Center, Dallas, Texas, USA;

[3] Texas A&M University, College Station, Texas, USA;

[4] Department of Ophthalmology, University of Texas Southwestern Medical Center, Dallas, Texas, USA;

[5] Department of Pediatrics, University of Texas Southwestern Medical Center, Dallas, Texas, USA;

[6] Department of Microbiology, University of Texas Southwestern Medical Center, Dallas, Texas, USA;

[7] Department of Molecular Biophysics, University of Texas Southwestern Medical Center, Dallas, Texas, USA;

[8] Faculty of Engineering and Natural Sciences, Sabanci University, Istanbul, Turkey;

[9] Department of Pharmacology, University of Texas Southwestern Medical Center, Dallas, Texas, USA;

*e-mail: erdal.toprak@utsouthwestern.edu

Supporting Figures/Tables

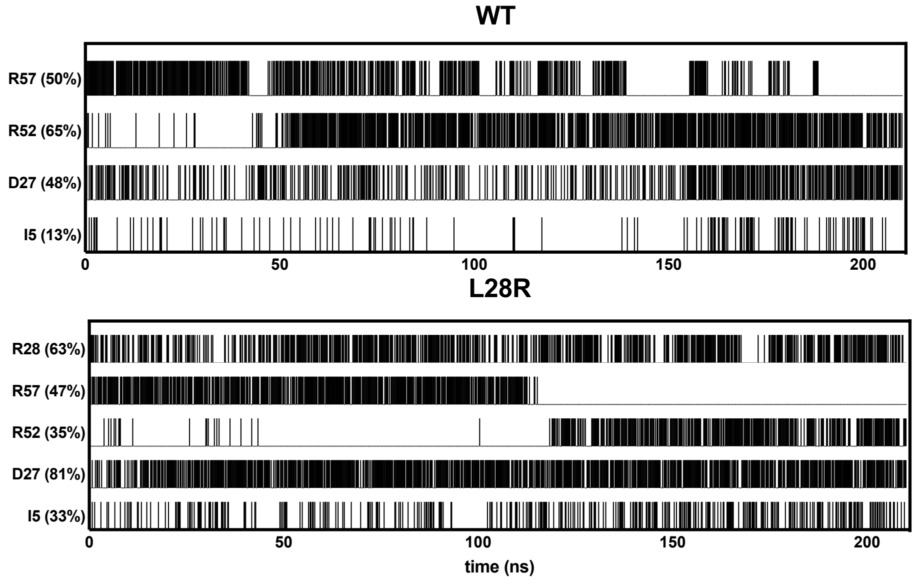

Supplementary Figure 1| Barcode graphs for the hydrogen bonds established between binding cavity residues and folate in the MD trajectories for the wild-type and L28R DHFR. Value is 1 if there is at least 1 hydrogen bond between any atom of the residue and folate, 0 otherwise; hydrogen bond cutoff distance is set at a moderate value of 3.2 Å. The details of the MD trajectories are given in Ref. Abdizadeh et al. 2017. Note how in the mutant, releasing the ligand contacts with R52 is followed by establishing those with R57.

Supplementary Table 1| Mean IC_{95} values of TMP and other molecules we tested against wild-type and L28R *E. coli* [1]

Entry	Compound	Molecular formula	Molecular weight	MIC against wild-type *E. coli* (μg/mL)	MIC against L28R *E. coli* (μg/mL)
1	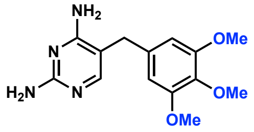 Trimethoprim (TMP)	$C_{14}H_{18}N_4O_3$	290.32	0.89 ± 0.1	932 ± 330
2	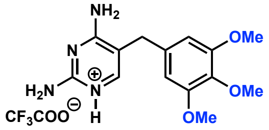 Trimethoprim trifluoroacetate salt	$C_{16}H_{19}F_3N_4O_5$	404.35	1.42± 0.21	1362±410
3	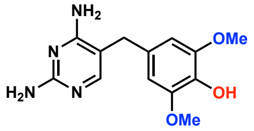 4'-Desmethyltrimethoprim (4'-DTMP)	$C_{13}H_{16}N_4O_3$	276.30	1.62 ± 0.25	40.2 ± 0.63
4	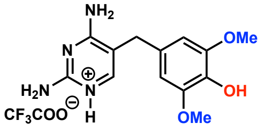 4'-Desmethyltrimethoprim trifluoroacetate salt	$C_{15}H_{17}F_3N_4O_5$	390.32	2.66 ± 0.28	60.0± 0.87
5	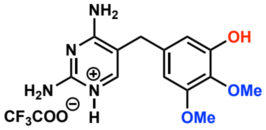 3'-Desmethyltrimethoprim trifluoroacetate salt	$C_{15}H_{17}F_3N_4O_5$	390.32	624.5 ± 33.2	>3512
6	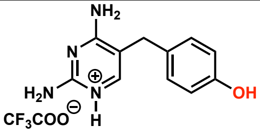	$C_{13}H_{13}F_3N_4O_3$	330.27	275.1 ± 9.0	2477 ± 21
7	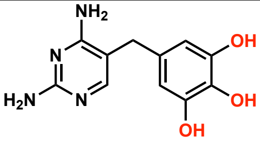	$C_{11}H_{12}N_4O_3$	248.24	160.0 ± 5.0	819.2 ± 17.8
8	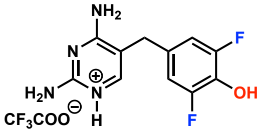	$C_{13}H_{11}F_5N_4O_3$	366.25	>3296	>3296
9	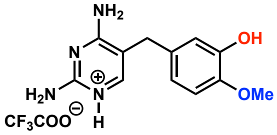	$C_{14}H_{15}F_3N_4O_4$	360.29	>3242	>3242

[1] n = 7 replicates; SEM = standard error on the mean. IC_{95} values of some of the compounds were not quantifiable as the cells survived the highest drug concentration we used. Several molecules had poor solubility in M9 media and therefore, we made trifluoroacetate salts to improve their solubility in aqueous media.

Supplementary Table 2| Mean IC_{95} values of TMP and 4'-DTMP against *E. coli* DHFR mutant library with all possible combinations of six frequently observed mutations[1].

Sl No.	Mutant	Mean IC95 (µg/mL) ± SEM		Sl No.	Mutant	Mean IC95 (µg/mL) ± SEM	
		TMP	4'-DTMP			TMP	4'-DTMP
1	MG1655*	0.4±0	1.1±0	47	P21L-A26T-L28R-W30G-I94L	3±0	1±0
2	WT	16±2	9±0	48	c-35t	97±0	31±0
3	I94L	76±17	24±5	49	c-35t-I94L	678±157	215±50
4	W30R	33±0	11±0	50	c-35t-W30R	871±0	93±0
5	W30R-I94L	162±52	65±22	51	c-35t-W30R-I94L	>2612	645±150
6	W30G	25±5	11±0	52	c-35t-W30G-I94L	>2612	829±0
7	W30G-I94L	>2612	133±60	53	c-35t-L28R	>2612	277±0
8	L28R	140±63	42±21	54	c-35t-L28R-I94L	>2612	215±50
9	L28R-I94L	871±0	93±0	55	c-35t-L28R-W30R	>2612	>2487
10	L28R-W30R	>2612	72±16	56	c-35t-L28R-W30R-I94L	>2612	645±150
11	L28R-W30G	>2612	93±0	57	c-35t-L28R-W30G	>2612	645±150
12	L28R-W30G-I94L	4±0	2±0	58	c-35t-L28R-W30G-I94L	>2612	645±150
13	A26T	76±17	15±6	59	c-35t-A26T	291±0	93±0
14	A26T-I94L	162±52	52±16	60	c-35t-A26T-I94L	>2612	645±150
15	A26T-W30R	162±52	93±0	61	c-35t-A26T-W30R	>2612	645±150
16	A26T-W30R-I94L	1452±474	399±180	62	c-35t-A26T-W30R-I94L	>2612	645±150
17	A26T-W30G	484±157	93±0	63	c-35t-A26T-W30G	>2612	829±0
18	A26T-W30G-I94L	0±0	0±0	64	c-35t-A26T-W30G-I94L	>2612	829±0
19	A26T-L28R	>2612	277±0	65	c-35t-A26T-L28R	>2612	645±150
20	A26T-L28R-I94L	>2612	829±0	66	c-35t-A26T-L28R-I94L	>2612	829±0
21	A26T-L28R-W30R	5±2	2±0	67	c-35t-A26T-L28R-W30R	>2612	829±0
22	A26T-L28R-W30R-I94L	3±0	1±0	68	c-35t-A26T-L28R-W30R-I94L	>2612	645±150
23	A26T-L28R-W30G	>2612	829±0	69	c-35t-A26T-L28R-W30G	>2612	645±150
24	A26T-L28R-W30G-I94L	25±5	4±0	70	c-35t-A26T-L28R-W30G-I94L	>2612	645±150
25	P21L	97±0	31±0	71	c-35t-P21L	484±157	154±50
26	P21L-I94L	97±0	31±0	72	c-35t-P21L-I94L	2033±474	277±0
27	P21L-W30R	97±0	31±0	73	c-35t-P21L-W30R	>2612	277±0
28	P21L-W30R-I94L	291±0	72±16	74	c-35t-P21L-W30R-I94L	>2612	829±0
29	P21L-W30G	226±52	52±16	75	c-35t-P21L-W30G	>2612	277±0
30	P21L-W30G-I94L	3±0	1±0	76	c-35t-P21L-W30G-I94L	38±24	2±0
31	P21L-L28R	484±157	31±0	77	c-35t-P21L-L28R	>2612	277±0
32	P21L-L28R-I94L	4±0	2±0	78	c-35t-P21L-L28R-W30R	>2612	645±150
33	P21L-L28R-W30R	3±0	1±0	79	c-35t-P21L-L28R-W30R-I94L	>2612	18±5
34	P21L-L28R-W30R-I94L	4±0	3±0	80	c-35t-P21L-L28R-W30G	>2612	645±150
35	P21L-L28R-W30G	3±0	1±0	81	c-35t-P21L-L28R-W30G-I94L	7±2	3±0
36	P21L-L28R-W30G-I94L	3±0	2±0	82	c-35t-P21L-A26T	1452±474	215±50
37	P21L-A26T	291±0	72±16	83	c-35t-P21L-A26T-I94L	>2612	645±150
38	P21L-A26T-I94L	226±52	31±0	84	c-35t-P21L-A26T-W30R	>2612	645±150
39	P21L-A26T-W30R	291±0	72±16	85	c-35t-P21L-A26T-W30R-I94L	>2612	645±150
40	P21L-A26T-W30R-I94L	3±0	1±0	86	c-35t-P21L-A26T-W30G-I94L	1±0	1±0
41	P21L-A26T-W30G-I94L	3±0	2±0	87	c-35t-P21L-A26T-L28R	>2612	645±150
42	P21L-A26T-L28R	>2612	65±22	88	c-35t-P21L-A26T-L28R-I94L	>2612	645±150
43	P21L-A26T-L28R-I94L	2±0	2±0	89	c-35t-P21L-A26T-L28R-W30R	>2612	645±150
44	P21L-A26T-L28R-W30R	7±2	2±0	90	c-35t-P21L-A26T-L28R-W30R-I94L	9±2	2±0
45	P21L-A26T-L28R-W30R-I94L	4±0	2±0	91	c-35t-P21L-A26T-L28R-W30G-I94L	4±0	2±0
46	P21L-A26T-L28R-W30G	3±0	2±0				

[1] n = 3 replicates; SEM = standard error on the mean. IC_{95} values of some of the mutants were not quantifiable as they survived the highest drug concentration we used (2612 µg/mL for TMP and 2487 µg/mL for 4'-DTMP). Hence, we report their IC_{95} values as >2612 µg/mL and >2487 µg/mL for TMP and 4'-DTMP, respectively. The summary of these experiments is shown in Supplementary Figure 2. *The IC_{95} values of MG1655 was measured in a different set of experiment. We note that the WT strain exhibits mild over-expression of DHFR likely because of the inserted selection markers with cloning and hence it has a higher IC_{95} value compared to MG1655, the standard laboratory WT strain.

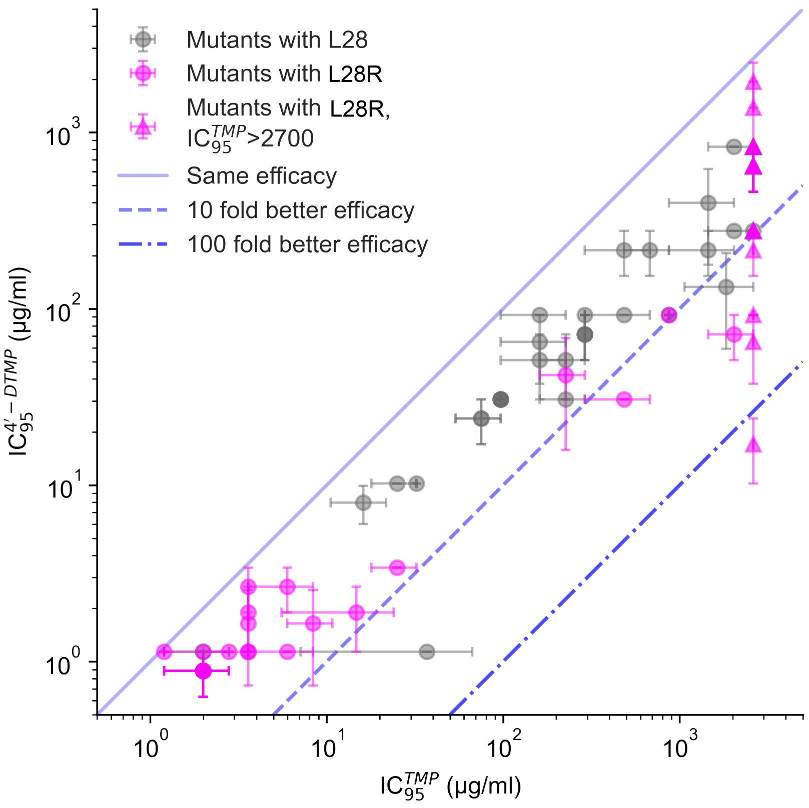

Supplementary Figure 2| Comparison of IC_{95} values of TMP and 4'-DTMP against *E. coli* DHFR mutant library with all possible combinations of six frequently observed DHFR mutations (c-35t, P21L, A26T, L28R, W30G, W30R, and I94L). We quantified resistance of these mutants against TMP and 4'-DTMP. Overall, 4'-DTMP had better efficacy in comparison to TMP against most of the *E. coli* mutant combinations (n = 3 replicates, error bars show the standard error on the mean, center of the error bars corresponds to the mean value of the measurements). Mutants with L28R replacement are represented with magenta markers. TMP addition did not stop the bacterial growth of many highly resistant *E. coli* mutants with L28R mutation, even at 2612 μg/mL concentration, whereas 4'-DTMP addition stopped the growth at concentrations several fold lower than concentration of TMP (magenta triangles). TMP resistance of these mutants (magenta triangle) are not quantifiable as they survive the highest TMP dose we used. Hence, we report these IC_{95} values as >2612 μg/mL.

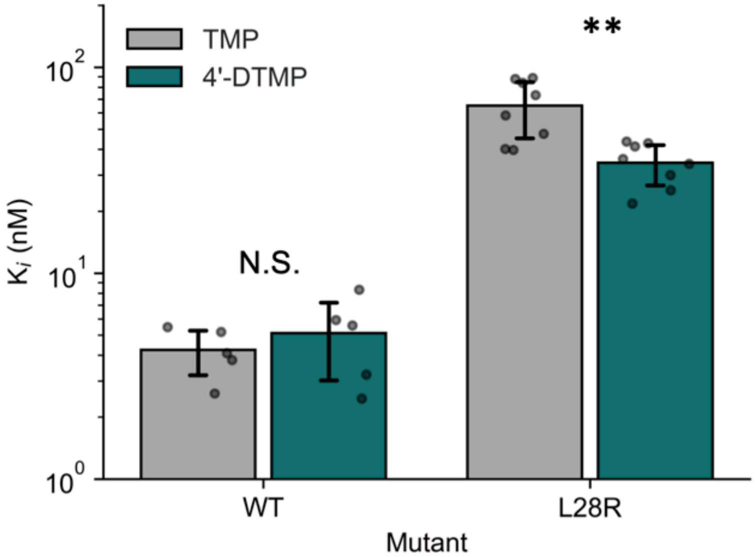

Supplementary Figure 3| Determination of *in vitro* binding affinities (K_i) of TMP and 4'-DTMP to $DHFR^{WT}$ and $DHFR^{L28R}$ using purified proteins. TMP (gray) and 4'-DTMP (teal) had indistinguishable binding affinities (K_i = 4.2 nM and 5.1 nM, respectively; p = 0.467) to $DHFR^{WT}$ (left, n = 5 replicates) whereas, 4'-DTMP (teal) has higher binding affinity compared to TMP (gray) to $DHFR^{L28R}$ (right, n = 9 replicates; K_i = 34.3 nM and 65.0 nM, respectively; p = 0.0018). Student's *t*-test (two tailed) is used to quantify significance of K_i value differences (*p<0.05, **p<0.01, and ***p<0.001, error bars show the standard deviation, center of the error bars corresponds to the mean value of the measurements).

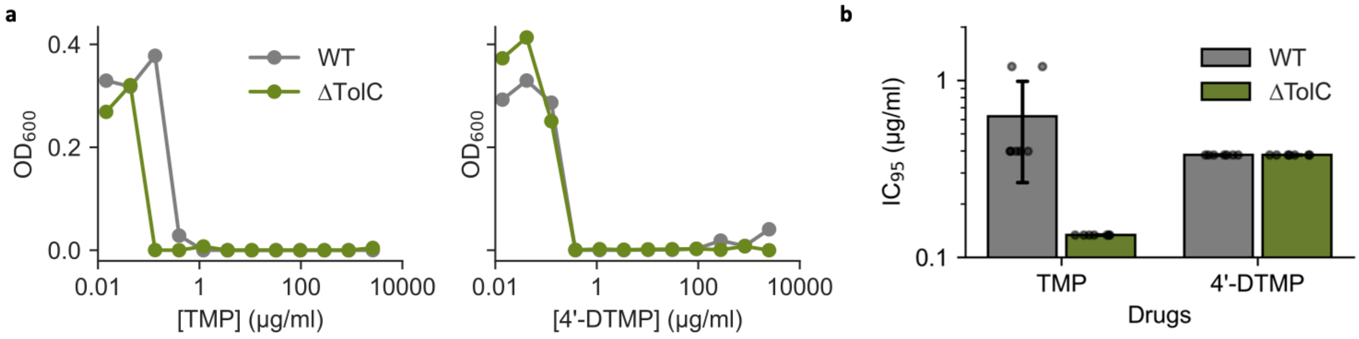

Supplementary Figure 4| TMP and 4’-DTMP have similar antibacterial efficacy against *E. coli* with (wild-type) or without (∆TolC) efflux machinery. a, Representative drug response curves for TMP (left) and 4’-DTMP (right) against wildtype (wild-type, gray) and ∆TolC variant (olive) of *E. coli*. **b**, TMP (left) has slightly enhanced activity against *E. coli*:∆TolC (olive) in comparison to wildtype (gray) whereas no change in activity was observed for 4’-DTMP (right) [n = 7 replicates, error bars show the standard deviation, center of the error bars corresponds to the mean value of the measurements]. These data indicate that TMP is marginally getting refluxed by wild-type *E. coli* but no efflux was observed in case of 4’-DTMP.

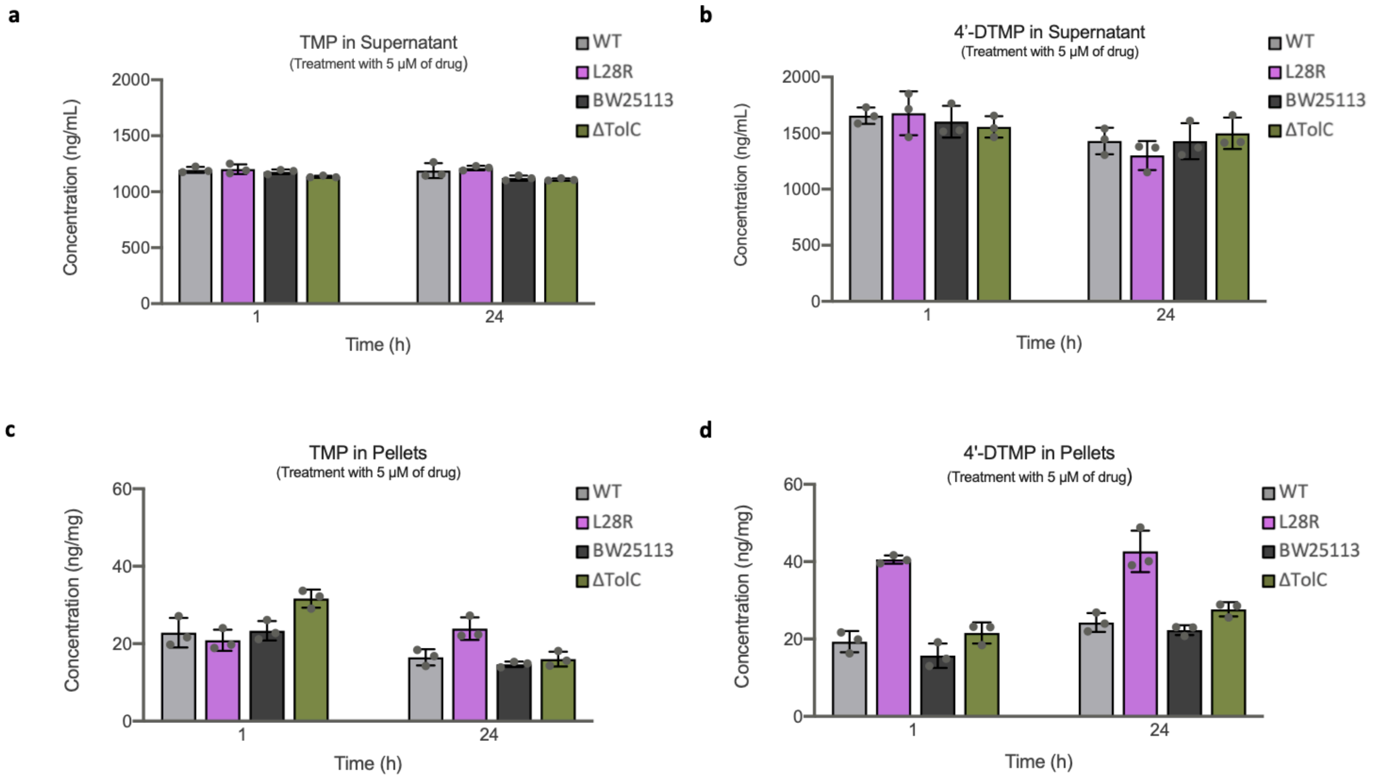

Supplementary Figure 5| Intracellular drug concentration measurement in wild-type, L28R, BW25113, and BW25113:∆TolC *E. coli* strains. TMP or 4’-DTMP were incubated at 5 μM (sub-MIC) concertation with wild-type, L28R, BW25113, and BW25113:∆TolC *E. coli* cells and then the resulting supernatant or cell fraction was measured for TMP or 4’-DTMP concentrations by LC-MS/MS after 1 h and 24 h (n = 3 replicates for each strain and each time point, error bars show the standard error in the mean, center of the error bars corresponds to the mean value of the measurements). 4’-DTMP was found to accumulate more in L28R *E. coli* cells compared to other *E. coli* strains. Also, for L28R *E. coli* cells, accumulation of 4’-DTMP was more compared to TMP in both the time points.

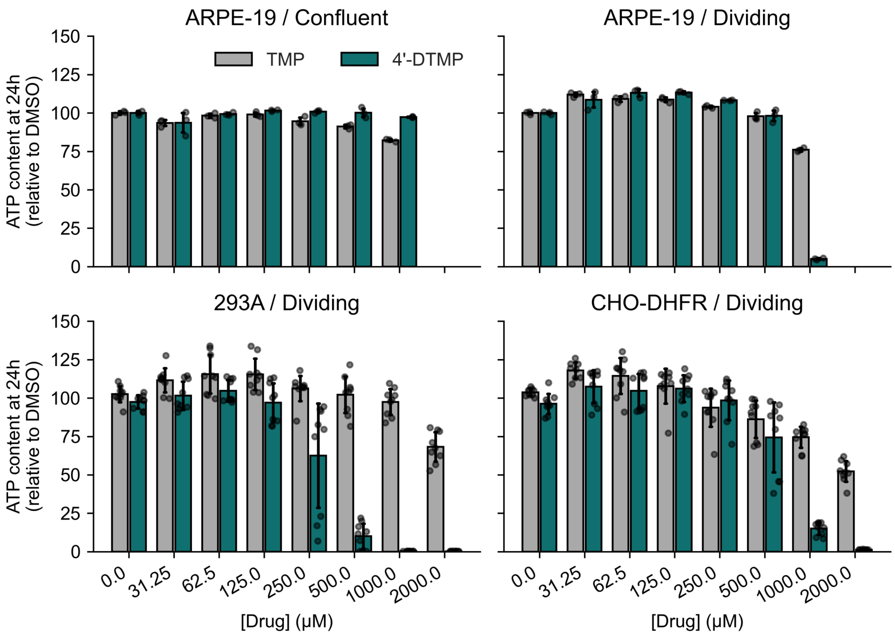

Supplementary Figure 6| Toxicity profiles of TMP and 4'-DTMP against confluent and diving human cells (ARPE-19 cells, HEK293A, and CHO-DHFR cells). ATP contents at 24 h for confluent (top left), diving (top right) ARPE-19, dividing HEK293A (bottom left), and dividing CHO-DHFR (bottom right) human cells are shown after the treatment with TMP (gray) and 4'-DTMP (teal) at different concentrations (n = 3 replicates for ARPE-19 cell line whereas, n = 8 replicates for 293A and CHO-DHFR cell line, error bars show the standard deviation, center of the error bars corresponds to the mean value of the measurements). Neither of the compounds are toxic up to 0.5 mM. At 1 mM, 4'-DTMP has toxicity against dividing cells.

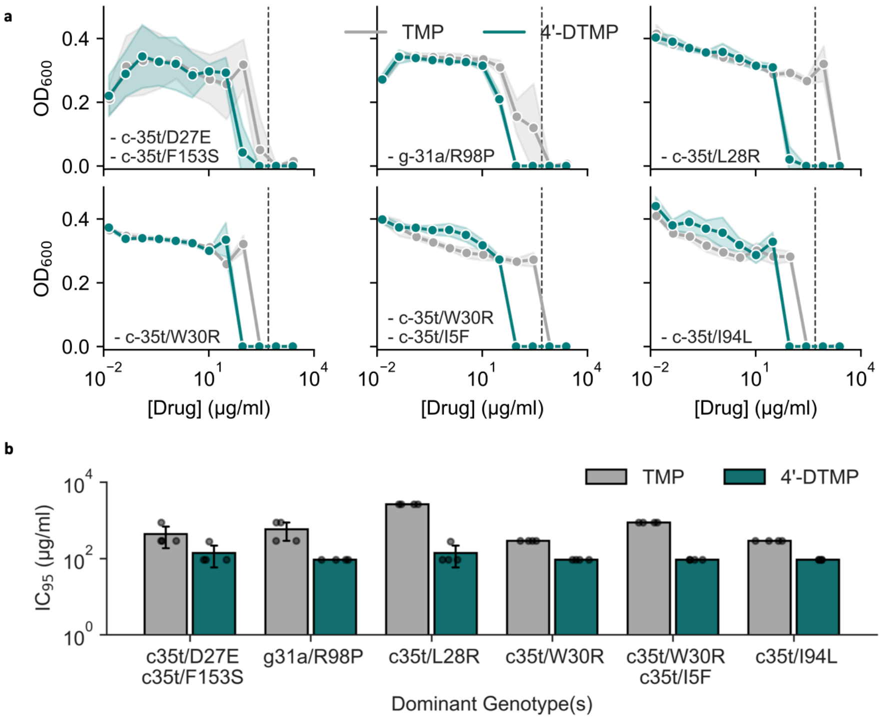

Supplementary Figure 7| Determination of optimum drug concentration required for propagation of mixed populations experiment. a, Overlaid drug-response curves for TMP (gray, n = 3 replicates) and 4'-DTMP (teal, n = 3 replicates) against *E. coli* populations with different DHFR mutants (obtained from our previous morbidostat evolution experiment under TMP selection). Lines with filled circles represent mean values and shaded areas represent standard error on the mean. Mutations listed in the legends are found by Sanger sequencing of the *folA* gene amplified from randomly selected colonies from these populations. **b**, IC_{95} values of TMP (gray) and 4'-DTMP (teal) against the corresponding *E. coli* populations (n = 4 replicates, error bars show the standard deviation, center of the error bars corresponds to the mean value of the measurements). A drug concentration of 500 μM (gray dotted line) was chosen for the mixed population experiment so that all the mutants have similar chances to survive the drug dose.

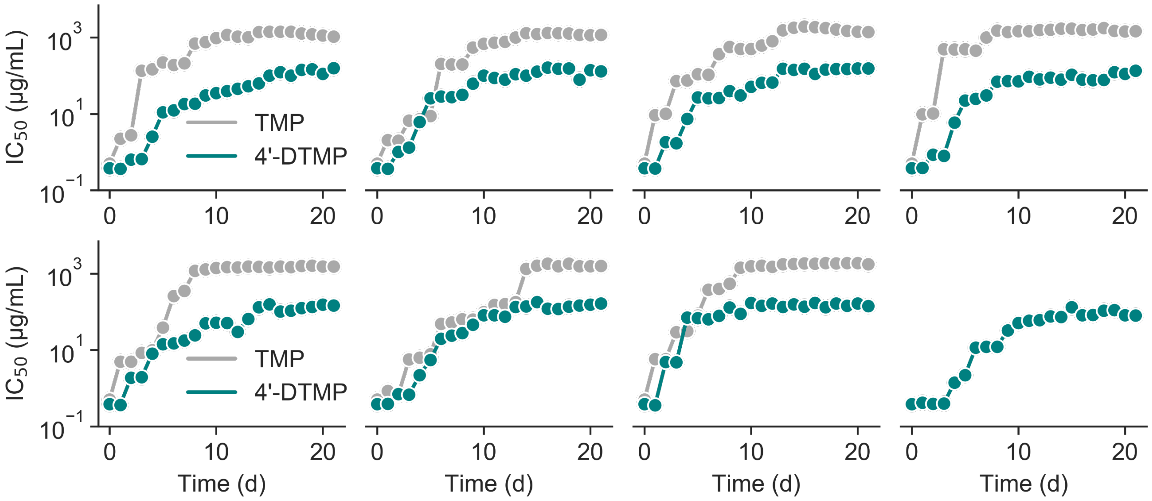

Supplementary Figure 8| L28R-specific 4'-DTMP slows down evolution of antibiotic resistance in the morbidostat. Changes in resistance levels (IC_{50}) over time for *E. coli* populations evolved in parallel under inhibition by 4'-DTMP (teal, n = 8 replicates) and TMP (gray, n = 7 replicates). Day 0 corresponds to ancestral strain.

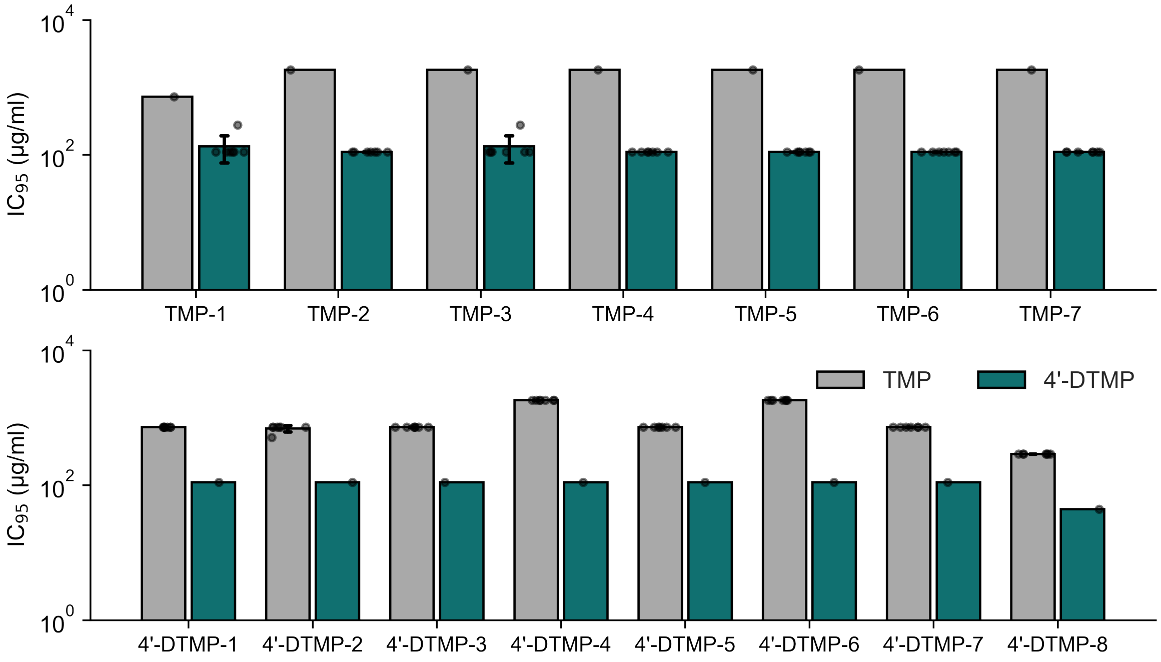

Supplementary Figure 9| IC_{95} values of TMP and 4'-DTMP against the individual *E. coli* populations (Day 21) evolved under TMP (top panel; TMP-1 through TMP-7) and 4'-DTMP (bottom panel; 4'-DTMP-1 through 4'-DTMP-8) [n = 7 replicates, error bars show the standard deviation, center of the error bars corresponds to the mean value of the measurements]**.** The populations evolved under TMP selection are relatively less resistant to 4'-DTMP whereas, the populations evolved under 4'-DTMP selection are found to be highly resistant to TMP.

Supplementary Table 3| Growth rates of individual *E. coli* populations evolved under TMP and 4'-DTMP (isolated at Day 21) in absence of any drug.[1]

Strains	Doubling time (min)				
	Replicate1	Replicate2	Replicate3	Replicate4	Average ± STD
**WT**	48.08<br>46.87	46.68<br>46.53	46.49<br>50.12	47.05<br>52.33	48.02±2.12
**TMP-1**	44.03	44.32	42.8	42.67	43.46±0.84** (p = 0.0023)
**TMP-2**	49.09	48.59	50.19	48.89	49.19±0.69
**TMP-3**	48.72	52.54	41.95	42.76	46.49±0.5.03
**TMP-4**	45.07	44.9	45.02	47.16	45.54±1.08
**TMP-5**	74.42	70.83	70.87	71.95	72.02±1.68*** (p = $2.657e^{-9}$)
**TMP-6**	41.2	43.87	46.757	45.907	44.43±2.47* (p = 0.0256)
**TMP-7**	49.72	51.72	42.71	45.44	47.40±4.07
**4'-DTMP-1**	87.03	87.95	83.35	80.22	84.64±3.55*** (p = $6.239e^{-10}$)
**4'-DTMP-2**	60.9	65.01	63.56	59.78	62.32±2.39*** (p = $9.586e^{-7}$)
**4'-DTMP-3**	89.33	83.47	80.39	76.34	82.38±5.48*** (p = $1.772e^{-8}$)
**4'-DTMP-4**	67.74	63.94	63.01	63.69	64.59±2.13*** (p = $1.681e^{-7}$)
**4'-DTMP-5**	46.02	48.32	50.39	51.04	48.94±2.27
**4'-DTMP-6**	57.72	58.38	58.02	58.74	58.21±0.44*** (p = $3.14e^{-6}$)
**4'-DTMP-7**	49.93	49.26	52.94	49.23	50.34±1.77
**4'-DTMP-8**	58.24	55.83	60.27	59.44	58.44±1.93*** (p = $9.19e^{-6}$)

[1] Student's *t*-test (two tailed) is used to quantify significance of doubling time of each strain compared to wild-type ancestor *E. coli* strain (WT) (*p<0.05, **p<0.01, and ***p<0.001). The strains evolved under 4'-DTMP had significantly slower growth rates compared to the strains evolved under TMP (average doubling time 63.7±12.5 min and 49.8±10 min, respectively; p = 0.04).

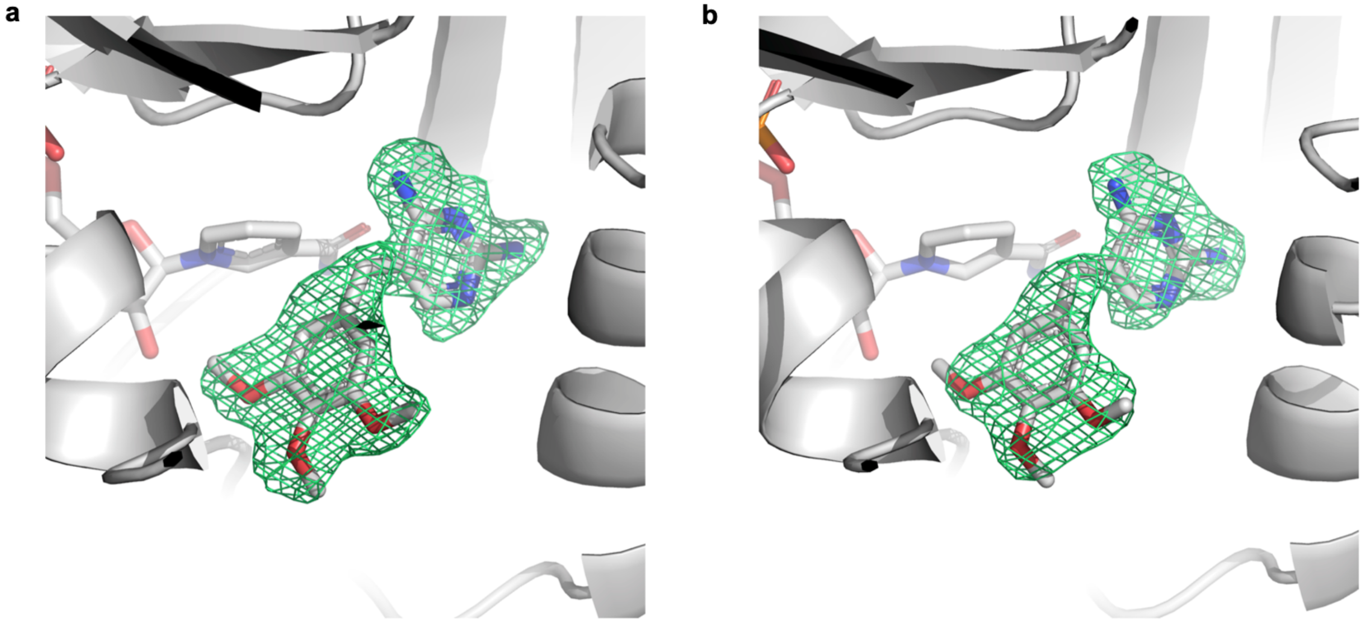

Supplementary Figure 10| Omit difference electron density maps (contoured at 3.5σ) for the ligand TMP in a) Wild-type DHFR structure (6XG5), and b) L28R DHFR structure (6XG4).

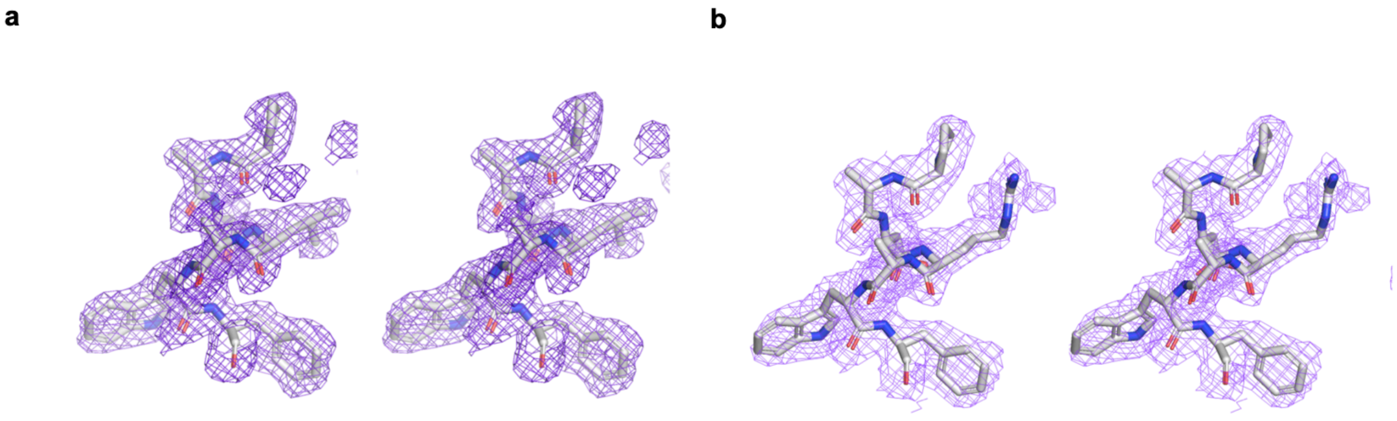

Supplementary Figure 11| Representative 2mFo-DFc electron density maps contoured at 1.5σ for fragments of the a) Wild-type DHFR structure (6XG5), and b) L28R DHFR structure (6XG4). The same fragment of the model is shown for both structures and it includes the site of mutation.

Supplementary Table 4| Primers used in this study

**Primer Name**	**Description**	**DNA Sequence**
FWD_P1	Portion 1 forward adapter	ACACTCTTTCCCTACACGACGCTCTTCCGATCTNNNNNNNNCAGCAGAATATAAAATTTTCC
REV P1	Portion 1 reverse adapter	GTGACTGGAGTTCAGACGTGTGCTCTTCCGATCTNNNNNNNNAACTGTTCATAAACGCGACC
FWD_P2	Portion 2 forward adapter	ACACTCTTTCCCTACACGACGCTCTTCCGATCTNNNNNNNNAATAAACCCGTGATTATGGG
REV P2	Portion 2 reverse adapter	GTGACTGGAGTTCAGACGTGTGCTCTTCCGATCTNNNNNNNNGCGTCGCATCCGGCGCTAGC

Supplementary Notes

Chemical methods

Unless otherwise indicated, all reactions were carried out in flame-dried glassware with magnetic stirring. Commercially obtained reagents were used as received. Solvents were dried by passage through an activated alumina column under argon. Liquids and solutions were transferred via syringe or measuring cylinder. All reactions were monitored by thin-layer chromatography with Merck silica gel 60 F254 pre-coated plates (0.25 mm). All flash chromatography purifications were performed on a Teledyne Isco CombiFlash® Rf unless otherwise indicated. Silica gel (particle size 0.032-0.063 mm) purchased from SiliCycle was used for flash chromatography. ^{1}H and ^{13}C spectra were recorded on Varian Inova-400 or 500 spectrometers. NMR spectra were visualized in MestReNova V14.0.1. Data for ^{1}H NMR spectra are reported relative to dimethyl sulfoxide as an internal standard (2.50 ppm) and are reported as follows: chemical shift (δ ppm), multiplicity, coupling constant (Hz), and integration. Data for ^{13}C NMR spectra are reported relative to dimethyl sulfoxide as an internal standard (39.5 ppm) and are reported in terms of chemical shift (δ ppm).

Supplementary Note 1: Preparation of 4'-DTMP

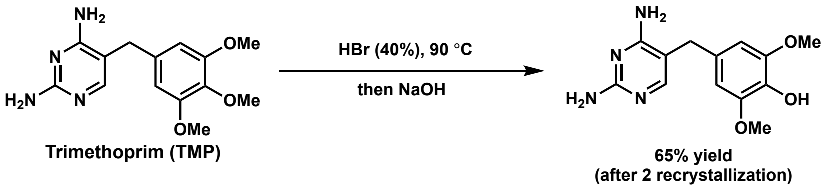

4'-Desmethyltrimethoprim (4'-DTMP, CAS No. 21253-58-7) was prepared according to a modified literature procedure[1]. In a 500 mL round-bottom flask, equipped with a reflux condenser, TMP (10.0 g, 34.44 mmol, Combi-Blocks, QA-3343) was taken and HBr (125.0 mL, 48 percent in water) was gently added to it. The resulting mixture was then refluxed at 95 °C for 45 min. The reaction flask was cooled to 50 °C, placed in an ice-bath and the pH of the mixture was adjusted to ~7 using dropwise addition of 50% aqueous NaOH solution (an exothermic reaction occurs, slow addition is recommended). The off-white precipitation formed was kept at 4 °C, overnight. The precipitation was filtered, collected and re-dissolved in boiling water and kept at 4 °C for 20 h. The precipitation was filtered, collected and recrystallized from boiling methanol to obtain 4'-DTMP as an off-white solid (6.2 g, 65% yield). ^{1}H-NMR (DMSO-D_6) δ 8.22 (br s, 1H), 7.50 (br s, 2H), 7.37 (s, 1H), 7.02 (br s, 2H), 6.54 (s, 2H), 3.71 (s, 6H), 3.52 (s, 2H). ^{13}C-NMR (DMSO-D_6) δ 163.9, 156.6, 148.4, 144.0, 134.5, 128.1, 109.1, 106.8, 56.4, 32.6. LRMS (APCI+) calcd. for $[C_{13}H_{17}N_4O_3]^+$ ($[M+H]^+$): 277.13, found 277.17.

Supplementary Figure 12| ^{1}H-NMR of 4'-DTMP

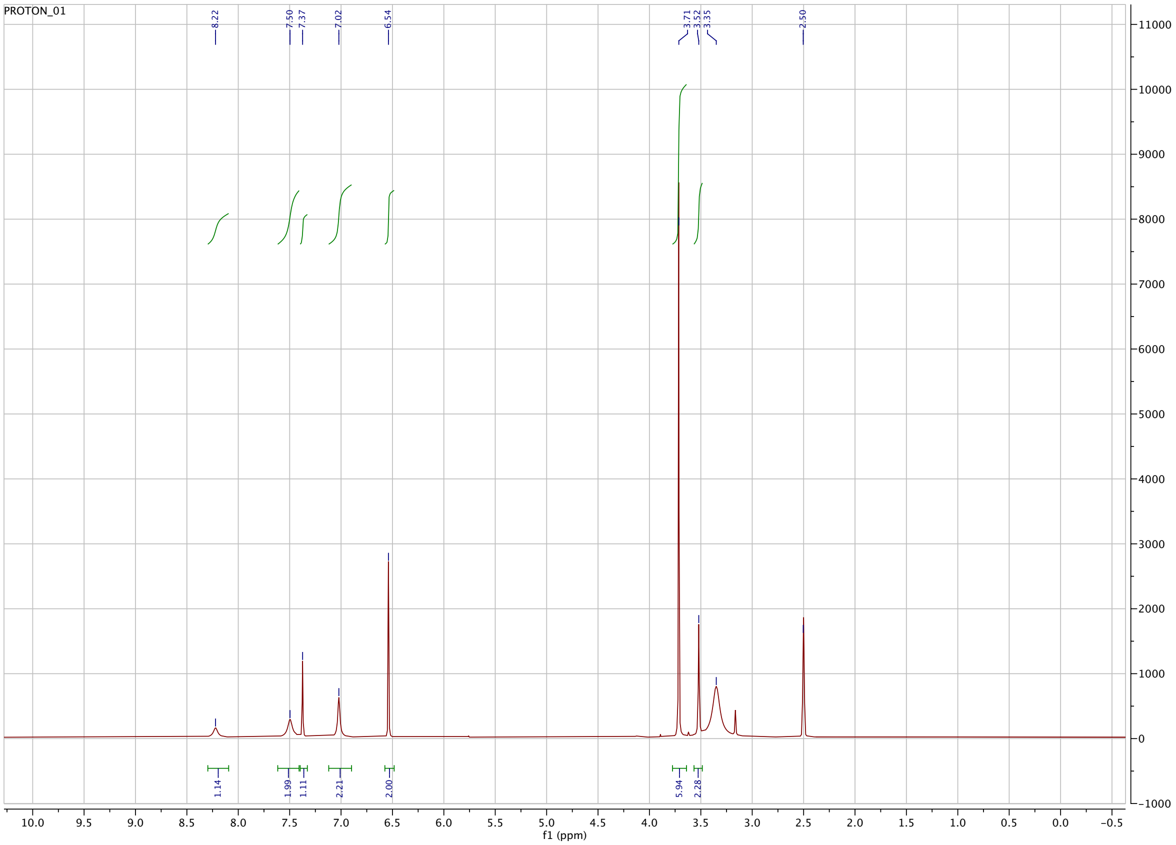

Supplementary Figure 13| ^{13}C-NMR of 4'-DTMP

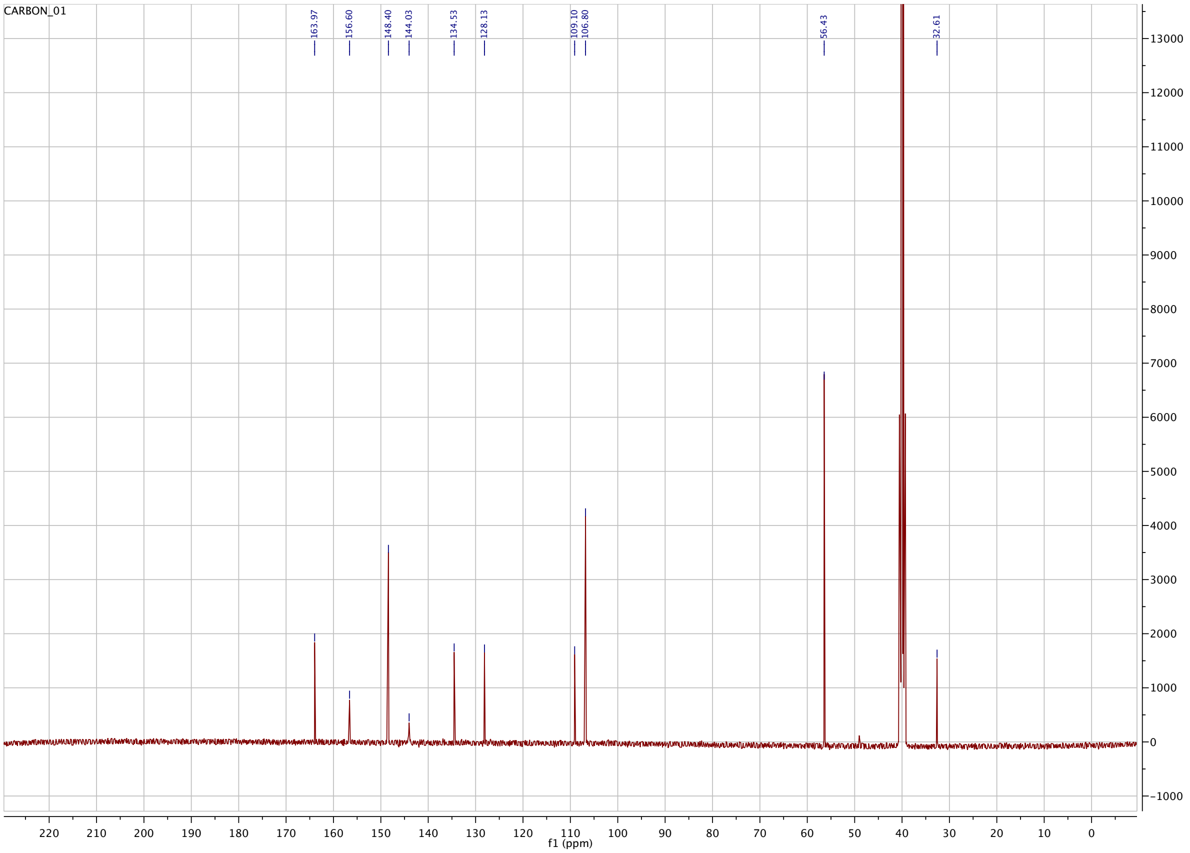

Supplementary References:

1 Calloway, N. T. *et al.* Optimized Fluorescent Trimethoprim Derivatives for in vivo Protein Labeling. *ChemBioChem* **8**, 767-774, doi:10.1002/cbic.200600414 (2007).
