# Supplementary material for: A trimethoprim derivative impedes antibiotic resistance evolution
Source: Nat Commun. 2021 May 19;12:2949. doi: 10.1038/s41467-021-23191-z (PMC8134463; doi:10.1038/s41467-021-23191-z)
Supplement: Supplementary file 2 — Reporting Summary [file 41467_2021_23191_MOESM2_ESM.pdf]

nature research

Corresponding author(s): Erdal Toprak

Last updated by author(s): 03/25/2021

Reporting Summary

Nature Research wishes to improve the reproducibility of the work that we publish. This form provides structure for consistency and transparency in reporting. For further information on Nature Research policies, see our Editorial Policies and the Editorial Policy Checklist.

Statistics

For all statistical analyses, confirm that the following items are present in the figure legend, table legend, main text, or Methods section.

n/a	Confirmed	
☐	☒	The exact sample size (n) for each experimental group/condition, given as a discrete number and unit of measurement
☐	☒	A statement on whether measurements were taken from distinct samples or whether the same sample was measured repeatedly
☐	☒	The statistical test(s) used AND whether they are one- or two-sided<br>*Only common tests should be described solely by name; describe more complex techniques in the Methods section.*
☐	☒	A description of all covariates tested
☐	☒	A description of any assumptions or corrections, such as tests of normality and adjustment for multiple comparisons
☐	☒	A full description of the statistical parameters including central tendency (e.g. means) or other basic estimates (e.g. regression coefficient) AND variation (e.g. standard deviation) or associated estimates of uncertainty (e.g. confidence intervals)
☐	☒	For null hypothesis testing, the test statistic (e.g. F, t, r) with confidence intervals, effect sizes, degrees of freedom and P value noted<br>*Give P values as exact values whenever suitable.*
☒	☐	For Bayesian analysis, information on the choice of priors and Markov chain Monte Carlo settings
☒	☐	For hierarchical and complex designs, identification of the appropriate level for tests and full reporting of outcomes
☐	☒	Estimates of effect sizes (e.g. Cohen's d, Pearson's r), indicating how they were calculated

Our web collection on statistics for biologists contains articles on many of the points above.

Software and code

Policy information about availability of computer code

Data collection	Varian Inova-400, Tecan Plate Reader Infinite M200, Perkin Elmer LAMBDA 650, AB Sciex 4000 QTRAP® mass spectrometer coupled to a Shimadzu Prominence LC, BioTek Synergy 2 plate reader, Invitrogen Qubit 4 Fluorometer, Illumina MiSeq.
Data analysis	MestReNova V14.0.1, HKL3000 v2.3.12, MatLab v9.7, Python v3.7.7, CCP4 v7.1, PyMol v2.4.1, REFMAC v5.8.0258, Molprobity v4.5.1, COOT v0.9.4.1, MOLREP v11.7.02, Prism v9.0.0.

For manuscripts utilizing custom algorithms or software that are central to the research but not yet described in published literature, software must be made available to editors and reviewers. We strongly encourage code deposition in a community repository (e.g. GitHub). See the Nature Research guidelines for submitting code & software for further information.

Data

Policy information about availability of data

All manuscripts must include a data availability statement. This statement should provide the following information, where applicable:

- Accession codes, unique identifiers, or web links for publicly available datasets
- A list of figures that have associated raw data
- A description of any restrictions on data availability

Data supporting the findings of this study are available within the paper and its Supplementary Information. The X-Ray crystal structures have been deposited to PDB with DOI: 10.2210/pdb6XG4/pdb (L28R) and DOI: 10.2210/pdb6XG5/pdb (wild-type). Corresponding raw data for each figure and table used in the manuscript and the supplementary files are accessible free of cost from GitHub (https://github.com/erdaltoprak-zz/NatureCommunication2021_Manna.git). The raw sequencing data are deposited to NCBI with accession code: PRJNA717019. Link to the source data files are listed in the “Source Data” file provided with this paper.

Field-specific reporting

Please select the one below that is the best fit for your research. If you are not sure, read the appropriate sections before making your selection.

☒ Life sciences ☐ Behavioural & social sciences ☐ Ecological, evolutionary & environmental sciences

For a reference copy of the document with all sections, see nature.com/documents/nr-reporting-summary-flat.pdf

Life sciences study design

All studies must disclose on these points even when the disclosure is negative.

Sample size	Sample sizes were determined based on experimental feasibility, cost, and sufficient statistical power.
Data exclusions	No data were excluded from the analyses.
Replication	All experiments were replicated with at least three biological replicates. All attempts of replication were successful.
Randomization	Not applicable in this study.
Blinding	Not applicable in this study.

Reporting for specific materials, systems and methods

We require information from authors about some types of materials, experimental systems and methods used in many studies. Here, indicate whether each material, system or method listed is relevant to your study. If you are not sure if a list item applies to your research, read the appropriate section before selecting a response.

Materials & experimental systems

n/a	Involved in the study	
☒	☐	Antibodies
☐	☒	Eukaryotic cell lines
☒	☐	Palaeontology and archaeology
☒	☐	Animals and other organisms
☒	☐	Human research participants
☒	☐	Clinical data
☒	☐	Dual use research of concern

Methods

n/a	Involved in the study	
☒	☐	ChIP-seq
☒	☐	Flow cytometry
☒	☐	MRI-based neuroimaging

Eukaryotic cell lines

Policy information about cell lines

Cell line source(s)	ARPE-19 cells (CRL-2302, ATCC, Manassas, VA), CHO-DHFR (CRL-9096, ATCC) and HEK293A (R70507, Life Technologies, Carlsbad, CA)
Authentication	Human ARPE-19 and HEK293A cell lines were verified by STR profiling (University of Arizona Genetics Core).
Mycoplasma contamination	ARPE-19, CHO-DHFR, and HEK293A cell lines were verified to be free of mycoplasma using the Universal Mycoplasma Detection kit (30-1012K, ATCC)
Commonly misidentified lines (See ICLAC register)	*Name any commonly misidentified cell lines used in the study and provide a rationale for their use.*
